# Reduced lipid metabolite abundance in human pancreatic cancer and matched serum samples following neoadjuvant FOLFIRINOX treatment

**DOI:** 10.1007/s11306-025-02388-z

**Published:** 2026-01-19

**Authors:** Manoj Amrutkar, Sander Johannes Thorbjørnsen Guttorm, Knut Jørgen Labori, Helge Rootwelt, Katja Benedikte Prestø Elgstøen, Ivar P. Gladhaug, Caroline S. Verbeke

**Affiliations:** 1 Department of Pathology, Division of Laboratory Medicine, Oslo University Hospital, Rikshospitalet, 0424 Oslo, Norway; 2 Department of Medical Biochemistry, Division of Laboratory Medicine, Oslo University Hospital, Oslo, Norway; 3 Institute of Clinical Medicine, Faculty of Medicine, University of Oslo, Oslo, Norway; 4 Department of Hepato-Pancreato-Biliary Surgery, Oslo University Hospital, Oslo, Norway; 5 Core Facility for Global Metabolomics and Lipidomics, Institute of Clinical Medicine, Faculty of Medicine, University of Oslo, Oslo, Norway

**Keywords:** Pancreatic cancer, Neoadjuvant chemotherapy, FOLFIRINOX, Global lipidomics, LC–MS

## Abstract

**Introduction:**

Exploiting the full potential of neoadjuvant treatment (NAT) in pancreatic ductal adenocarcinoma (PDAC) is hampered by the lack of biomarkers for treatment response. Dysregulated lipid metabolism has been suggested to promote PDAC growth and resistance to therapy.

**Objectives:**

To investigate lipid metabolic changes in PDAC following NAT.

**Methods:**

Cross-sectional study of mass spectrometry-based global lipidomic profiling of tumour tissue (n = 35) and paired serum samples (n = 35) from treatment-naïve (TN; n = 18) and neoadjuvant FOLFIRINOX-treated (NAT; n = 17) PDAC patients was conducted. Pre- and post-treatment CA 19–9 levels were available from 15 NAT patients. Differentially abundant lipids (DALs) in NAT versus TN were assessed for correlation with various clinical parameters and the performance of all serum DALs to distinguish NAT from TN samples was explored using receiver operating characteristic analysis.

**Results:**

A total of 40 tissue and 35 serum DALs were identified, which mainly belonged to glycerophospholipids and sphingolipids in tissue and glycerolipids, glycerophospholipids, and fatty acyls in serum. All 19 serum glycerolipids were less abundant in NAT and 18 of these were triacylglycerols. The abundance of 26 tissue and 11 serum DALs correlated moderately with % reduction in serum CA 19–9 following NAT. The top five of 23 serum DALs with moderate discriminatory potential (AUC = 0.66–0.87) ‒ PI(18:0_20:3), AcCa(13:0), PC(O-42:6), TG(49:6), TG(66:14), performed better together (AUC = 0.93 and 95% CI = 0.79‒1) and combined with CA 19–9 (AUC = 0.99 and 95% CI = 0.81‒1).

**Conclusions:**

Both tumour tissue and serum samples from PDAC patients showed lower abundance of lipid metabolites following neoadjuvant FOLFIRINOX treatment. Moreover, a biomarker panel of CA 19–9 together with five serum DALs could potentially be used to assess NAT response in PDAC but requires further validation.

**Supplementary Information:**

The online version contains supplementary material available at 10.1007/s11306-025-02388-z.

## Introduction

Pancreatic ductal adenocarcinoma (PDAC) accounts for more than 90% of all pancreatic neoplasms. It has a 5-year survival rate of less than 10% and is expected to become the second leading cause of cancer-related death in the Western world by 2030 (Siegel et al. 2022; Rahib et al. 2014). Surgery is currently the only potentially curative treatment of PDAC, however, most patients (> 80%) are inoperable at diagnosis (Park et al. 2021). A multimodal approach consisting of neoadjuvant (preoperative chemotherapy) and/or adjuvant chemotherapy and surgery has been considered a possible treatment option for all non-metastatic PDACs (Park et al. 2021). As such, neoadjuvant treatment (NAT) has become a standard of care for borderline and locally advanced PDAC owing to its potential benefits related to the reduction of tumour burden, control of metastasis, and improved selection of patients eligible for surgical resection (Springfeld et al. 2023; Gugenheim et al. 2022; Farnes et al. 2023). Combination chemotherapy regimens including gemcitabine/nab-paclitaxel (Abraxane) and FOL()FIRINOX [leucovorin calcium (folinic acid), fluorouracil, irinotecan hydrochloride and oxaliplatin] are the standard options for NAT in PDAC (Conroy et al. 2011; Von Hoff et al. 2013; Conroy et al. 2023). Response to NAT in PDAC is currently evaluated preoperatively based on imaging and serum carbohydrate antigen 19–9 (CA 19–9) levels and postoperatively by histological assessment of tumour regression (Springfeld et al. 2023; Gugenheim et al. 2022; Holm et al. 2024). However, these methods do not accurately identify whether NAT is effective, and neither do they assess NAT effect throughout the course of treatment, thus missing out on opportunities to change from ineffective to potentially more effective treatment alternatives.

The molecular dynamics of PDAC in response to chemotherapy are poorly understood. Altered metabolic pathways fulfilling the energy needs of rapidly proliferating PDAC cells have emerged as a significant contributor to chemotherapy resistance (Qin et al. 2020; Tuerhong et al. 2021). Moreover, our recent multiomics studies revealed altered PDAC metabolic profiles in response to NAT. First, proteomic profiling revealed altered expression of metabolic pathway proteins in NAT versus treatment-naïve (TN) PDAC, both in tumour tissue and serum samples (Amrutkar et al. 2023). Second, metabolomic profiling revealed reduced abundance of metabolites associated with amino acid and nucleotide metabolism in neoadjuvant FOLFIRINOX-treated versus TN PDACs (Amrutkar et al. 2025). Moreover, NAT-induced downregulation of glycolysis was recently revealed by metabolic flux analysis (Tang et al. 2023). These studies suggest that the search for metabolites that are altered in response to NAT is an obvious way to identify potential markers of treatment response.

Metabolomics has been mainly used for the discovery of biomarkers for early detection and prediction of patient outcome in PDAC (Kaoutari et al. 2021; Zhao et al. 2023; Teng et al. 2024; Irajizad et al. 2023; Guo et al. 2022). It is only recently that metabolomics has been explored for predicting response to NAT in breast cancer and PDAC (Amrutkar et al. 2025; Diaz et al. 2022; Fang et al. 2025). Metabolomics mainly detects polar and amphipathic, water-soluble metabolites such as sugars, amino acids, organic acids and nucleotides, while it fails to identify most hydrophobic metabolites including lipids (Wang et al. 2020; Belhaj et al. 2021). Lipids are important biomolecules with structural, metabolic and signalling functions involved in the regulation of diverse physiological processes. Moreover, growing evidence suggests an association between altered lipid metabolic pathways and progression of PDAC (Yin et al. 2022; Wu et al. 2024). Hence, a complete profiling of lipid metabolites present in PDAC tissues and blood samples is of interest. It can be achieved by mass-spectrometry (MS)-based lipid profiling, an advanced branch of metabolomics that is referred to as lipidomics. Global, or untargeted lipidomics has the potential to comprehensively identify and quantify all lipid molecular species in a biological sample (Han 2016; Zullig et al. 2020 ; Liebisch et al. 2013) and has emerged as a promising tool for understanding disease mechanisms in PDAC as well as for early detection (Wolrab et al. 2022; Naudin et al. 2023).

Here, we performed mass spectrometry-based global lipidomic profiling of tumour tissue and matched serum samples obtained from PDAC patients who underwent surgical resection upfront (TN) or following neoadjuvant FOLFIRINOX (NAT) treatment. This proof-of-concept study characterizes chemotherapy-induced changes in PDAC lipid metabolic profile with the aim of identifying potential markers of NAT response in PDAC.

## Materials and methods

### Patients and samples

The study series consisted of 35 PDAC patients who underwent surgical resection at Oslo University Hospital, Rikshospitalet, Oslo, Norway, in the years 2015–2022. This is the same cohort as described in our recent study (Amrutkar et al. 2025). Of these, 17 were treated with neoadjuvant FOLFIRINOX (NAT group) followed by surgery, while 18 received upfront surgery (TN group). The collection of tumour tissue and matched serum samples was carried out as described previously (Amrutkar et al. 2023; Amrutkar et al. 2025). Briefly, tissue samples were collected and snap-frozen within 20 min of surgical resection. Blood samples collected the day before surgery were processed for serum extraction following standard procedures established at the hospital. Both tissue and serum samples were stored at − 80 °C until further use.

### Histopathological assessment

Histopathological assessment of the tissue block corresponding to the tumour bed, from which the omics sample was derived, was conducted as described previously (Amrutkar et al. 2025). Briefly, the surgical specimen was fixed in 10% neutral-buffered formalin for 48 h and examined by an experienced pancreatic pathologist (Verbeke et al. 2021). Tumour regression grading (TRG) for neoadjuvantly-treated PDACs was determined according to the College of American Pathologists (CAP) guidelines (Lawrence 2020).

### Chemicals

Water (type 1; > 18 MΩ cm) was obtained from MilliQ ultrapure water purification system (Merck Millipore, Darmstadt, Germany). Methanol was purchased from Rathburn Chemicals (Walkerburn, Scotland), acetonitrile (ACN) and formic acid (LC–MS grade) from Thermo Scientific Chemicals, methyl tert butyl ether (MTBE) and ammonium formate from Sigma-Aldrich (Steinheim, Germany), 2-propanol (IPA) from VWR Chemicals Bergen, Norway.

### Total lipid extraction

Prior to Liquid Chromatography-High Resolution Mass Spectrometry (LC-HigResMS or LC–MS), samples were processed as described previously (Amrutkar et al. 2025). For tissue samples, volumes of all chemicals were adjusted to tissue weight. Wherever applicable, samples were vortexed for 20 s at room temperature (RT) and centrifuged at 4 °C, 20,000 g for 10 min. Tissue samples were homogenized and mixed with methanol (15 µL/mg) and water (6 µL/mg), vortexed and centrifuged. Next, MTBE (15 µL/mg) and water (7.5 µL/mg) were added, vortexed, and centrifuged. For phase separation, samples were placed on ice for 5 min and subsequently in the refrigerator (4 °C) for 48 h. Next, chloroform (15 µL/mg) was added, and samples vortexed, centrifuged and kept at RT for 30 min for phase separation. Top and bottom phases were transferred to one Eppendorf tube for global lipidomic analysis, while the middle phase was transferred to a second tube for global metabolomic analysis, as reported previously (Amrutkar et al. 2025). Next, 250 µL of the phase mix for lipidomic analysis was dried under a gentle stream of nitrogen, and 200 µL IPA was added and mixed thoroughly. Serum samples were first thawed on ice. Next, 90 µL of cold IPA was added to 30 µL of serum, and samples were subsequently mixed, vortexed, and centrifuged. The supernatant was collected for LC–MS analysis. For each sample group (tissue and serum), separate pooled quality control (PQC) samples were prepared by transferring a 30 µL aliquot of each sample in the respective group into a separate Eppendorf tube. Lipid extraction of all samples was done in random order, independent of group.

### Lipid analysis

All processed samples including PQCs were collected in separate HPLC-vials with insert glass and cap for LC–MS analysis. All samples were analysed in random sequence independent of group. Tissue lipid extracts and serum lipid extracts were analysed in separate batches, which were analysed immediately following each other. Samples were analysed using Vanquish Horizon HPLC coupled to a Fusion Orbitrap tribrid mass spectrometer (Thermo Scientific) as described by Guttorm et al. (2025). Samples were analysed in both positive and negative ionization modes (in separate injections). For separation of lipids an Accucore C30 column (150 × 2.1 mm, 2.6 µm) was used with stepwise gradient elution, mobile phase A: ACN (60%) + H_2_O (40%) + 2 mM ammonium formate and mobile phase B: IPA (85.5%) + ACN (9.5%) + H_2_O (5%) + 2 mM ammonium formate. Full MS (of intact molecules for quantification) and MS/MS (fragmentation for lipid identification) analysis were performed, and samples were analysed in a randomized order following an analysis sequence setup as described by Skogvold et al. (2025). No internal standards were added to the samples.

### Data extraction and processing

Data were extracted and processed using Compound Discoverer 3.3.1 (Thermo Scientific) (Souza and Patti 2021). Instrumental drift was corrected using the SERRF-QC algorithm, available in Compound Discoverer. Features with a relative standard deviation > 30% in the PQC samples, and all features detected in the blank samples were excluded. Missing values were imputed using the “fill gaps” function which is included as a workflow node in the Compound Discoverer software. If a feature was detected in both the blank and study sample, a sample-to-blank ratio of ≥ 5:1 was required for it to be included. Only features with an intensity > 100,000 cps (counts per second) and a retention time between 0 and 24 min were included. Features with poor peak integration in the study samples were also excluded. It being a global analysis, many non-lipid features were also detected, including pharmaceuticals, plastics, and more polar metabolites. All features identified applying the inclusion/exclusion criteria were selected for further analysis. None of these were non-biological features, such as plastic or pharmaceuticals, that showed significantly altered levels between the comparing groups.

Unsupervised principal component analysis (PCA) was used to evaluate the analytical quality of analysis, to visualize the overall lipid profile of individual samples as well as variations among the samples and sample groups. The *p*-value per group ratio using a two-tailed student’s t-test was calculated with Compound Discoverer. Both treatment groups were compared using differential analysis in the form of volcano plots. Differentially abundant lipid (DAL) features with *p* < .05 and log2 fold change of < 0.5 or > 1 was considered statistically significant. *p*-values were adjusted for Benjamini–Hochberg correction to account for false discovery rate (FDR). DALs were annotated according to their confirmed structure match to in-house reference standards, LipidSearch 5.1 (Thermo Scientific) or tentatively to public databases, as described previously (Schymanski et al. 2014; Liebisch et al. 2013).

Lipidomics data for DALs (normalized peak area) were further explored and analysed using LipidOne 2.2 (Alabed et al. 2024; Pellegrino et al. 2022) and Qlucore Omics Explorer 3.8.22 (Qlucore AB, Lund, Sweden). For LipidOne following parameters were applied: the nomenclature‒sum composition, peak areas as the unit of measure, lipid class were selected as a feature, and groups were set to TN as a control and NAT as an experimental group. Correlation between lipid abundance and clinical parameters, including overall survival (OS) and change in serum CA 19–9 levels for NAT, was assessed using non-parametric Spearman’s correlation test. Correlation coefficient (r) values of 0 to (+/−)0.3, +/− (0.3 to 0.7), and +/− (0.7 to 1) were considered weak, moderate, and strong positive/negative correlation, respectively. Correlations with*p* < .05 were considered relevant. Heatmaps were generated using LipidOne, and scatter plots were generated using Qlucore Omics Explorer.

The MSI peak areas of all serum DALs and CA 19–9 values obtained from standard clinical chemistry analysis were submitted to MetaboAnalyst 6.0 (Pang et al. 2024) to evaluate their performance prediction as potential biomarkers. Univariate and multivariate receiver operating characteristic (ROC) analysis was used to assess the accuracy of model predictions through determination of specificity and sensitivity, as well as by comparing the area under the curve (AUC) of individual DALs and their combination using binary logistic regression, with the level of statistical significance (t-test) set at*p* < .05. ROC curves were generated using build-in functions for supervised classification algorithms support vector machine (SVM) classifiers available in LipidOne and MetaboAnalyst. Multivariate ROC exploratory analysis was used to identify promising biomarkers with high sensitivity and specificity.

## Results

### Characteristics of study participants

**Fig. 1:**
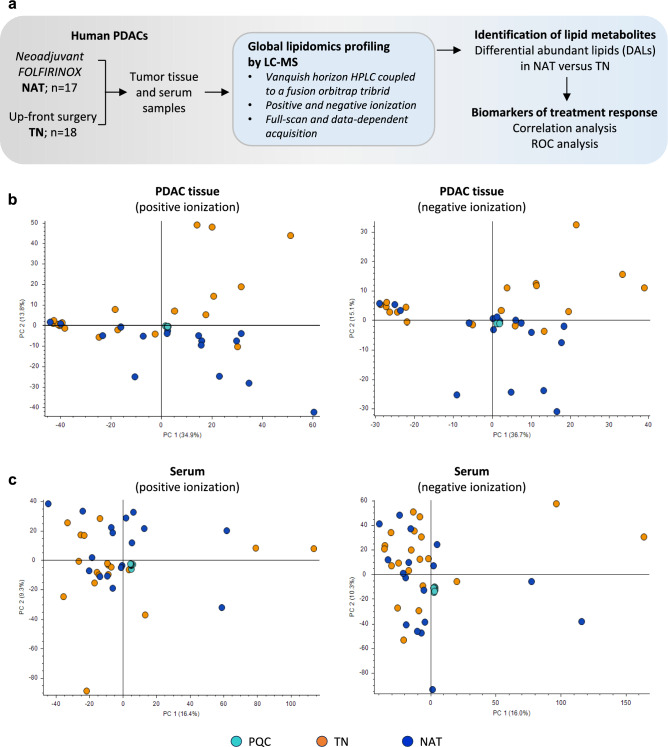
Study workflow and principal component analysis (PCA). **a** The study workflow for global lipidomic profiling of tumor tissue and paired serum samples from 17 neoadjuvant FOLFIRINOX-treated (NAT) and 18 treatment-naïve (TN) pancreatic ductal adenocarcinoma (PDAC) patients. (**b, c**) PCA plots showing distribution of tissue (**b**) and serum samples (**c**), including pooled quality controls (PQCs). X- and Y-axis represent component 1 and 2, respectively. HPLC, high-performance liquid chromatography; LC–MS, liquid chromatography mass spectrometry; FOLFIRINOX, folinic acid, 5-fluorouracil, irinotecan hydrochloride and oxaliplatin; ROC, receiver operating characteristics

The study workflow is shown in Fig. 1a. Of the 35 patients, 17 received neoadjuvant FOLFIRINOX (NAT group) and 18 underwent up-front surgery (TN group; Table 1**, **Table S1). All TN and 6 NAT patients had primary resectable, whereas 11 of 17 in the NAT group had borderline resectable or locally advanced cancer. Compared to TN, patients in the NAT group were younger (median age: 64 vs 74.5 years, *p* < .05) and had fewer comorbidities (47 vs 83%; Table 1). Serum CA 19–9 levels were 5.2-fold lower in NAT samples following chemotherapy (56 U/mL) compared to both pretreatment NAT samples (289 U/mL) and preoperative TN samples (290 U/mL), but this difference was not statistically significant. Histological assessment revealed moderate to poor response to NAT, corresponding to CAP-2 (n = 8) and CAP-3 (n = 9; Table 1). Adjuvant chemotherapy was administered to half of the patients in the TN group and to almost all patients in the NAT group (16 of 17). Median OS, calculated from the date of diagnosis, was 20.0 and 20.7 months for patients in NAT and TN group, respectively (Table 1).

**Table 1:** Clinical characteristics of the study population

Category	Treatment-naïve (TN, n = 18)	Neoadjuvantly-treated (NAT, n = 17)
**Gender**		
Male	13 (72%)	07 (41%)
Female	05 (28%)	10 (59%)
**Age** (years)	74.5 (50.0–84.0)	64.0 (51.0–75.0)*
**BMI** (kg/m^2^)	23.8 (20.7–39.6)	24.7 (17.3–34.8)
**Comorbidity**	15/18 (83%)	08/17 (47%)
**Disease stage**		
Primary resectable	18 (100%)	06 (35%)
Borderline resectable	–	10 (59%)
Locally advanced	–	01 (06%)
**Resection type**		
PPPDE/PDE	14 (78%)	16 (94%)
TP	00	01 (06%)
DP	04 (22%)	00 (00%)
**Tumor size** (mm)	37.0 (28.0–102.0)	35.0 (22.0–54.0)
**TNM classification** (8th edition) Tumor (T)		
T2	05 (28%)	07 (41%)
T3	13 (72%)	10 (59%)
**Lymph node metastasis** (N)		
N0	04 (22%)	02 (09%)
N1	05 (28%)	09 (41%)
N2	09 (50%)	11 (50%)
**Tumor regression grade**		
CAP 2	–	09 (53%)
CAP 3	–	08 (47%)
**Serum parameters** (preoperative)		
CA 19–9 (U/mL)	290.5 (11.0–11,371.0)	56.0 (7.0–477.0)
Bilirubin (µmol/L)	30.0 (5.0–341.0)	5.0 (3.0–24.0)*
Albumin (mg/dL)	40.5 (34.0–45.0)	42.0 (24.0–44.0)
C-reactive protein (mg/L)	4.3 (0.6–30.0)	2.4 (0.8–17.0)#
**Adjuvant treatment**	09/18 (50%)	16/17 (94%)
**Overall survival** (months)	20.7 (2.4–102.9)	20.0 (8.1–64.2)

Age, BMI, tumor size, serum parameters, and survival data are presented with median values and min–max range, while the data for all other categories is presented in actual numbers and percentage. Serum parameters values are from measurements done day before surgery. Overall survival (OS) was calculated from date of diagnosis. BMI, body-mass index; CA 19–9, Carbohydrate 19–9 antigen; CAP, College of American Pathologists; DP, distal pancreatectomy; PPPDE, pyloruspreserving pancreatoduodenectomy; TNM, tumor-node-metastasis; TP, total pancreatectomy. *p < .05 and ^#^p < .1, when compared between TN and NAT

### Principal component analysis

Total ion chromatograms (TIC) of PQC samples for both tissue and serum samples showed good peak distribution and high normalization level (NL) for each matrix (Fig. S1). Representative drift plots for one lipid per class are provided in Fig. S2a. The TIC obtained in negative ionization mode showed higher abundance of early eluting peaks, the NL was slightly lower compared to the positive ionization mode as expected. Following data pre-processing, the overall distribution of lipid features detected and their dispersion among all samples in both treatment groups and in relation to the PQCs was visualized using unsupervised PCA (Fig. 1b, c). The PCA plots were generated based on all lipid metabolite features detected by LC–MS and their respective peak areas in each individual sample. The PCA plots showed tight clustering of PQCs for both tissue and serum samples, demonstrating high analytical quality (Fig. 1b, c). The PCA plots for both tissue and serum displayed heterogeneity among samples within the same treatment group (TN and NAT) as well as between the groups. Interestingly, the component PC-2 appeared to separate most tissue samples between treatment groups (Fig. 1b). The PCA plots showed no obvious pattern that distinguished serum samples between treatment groups, irrespective of the ionization mode (Fig. 1c).

### Differentially abundant lipid (DAL) species in NAT versus TN group

**Fig. 2:**
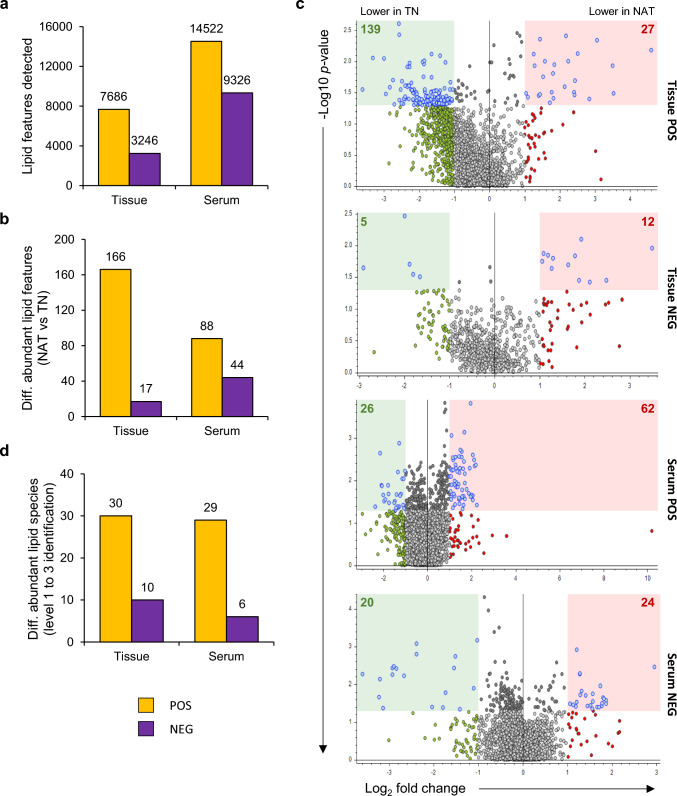
Overview of lipidomics profiles of PDAC tissue and serum samples. (**a**) Overview of all lipid features found in tissue and serum samples in both positive (POS) and negative (NEG) ionization modes. (**b**) Number of differentially abundant lipid (DAL) features in NAT versus TN group in both tissue and serum samples in both ionization modes. (**c**) Volcano plots showing distribution of DAL features according to their higher abundance in the respective treatment group. (**d**) Overview of DALs in NAT versus TN for both tissue and serum samples in both ionization modes. NAT, neoadjuvant FOLFIRINOX-treated; PDAC, pancreatic ductal adenocarcinoma; TN, treatment naïve

Total lipid features identified by MS across both ionization modes amount to 10,932 for tissue and 23,848 for serum samples (Fig. 2a). Most of these were detected in the positive ionization mode: 70.3% for tissue and 60.9% for serum samples. Only 183 lipid features in tissue and 132 in serum samples showed differential abundance (*p* < .05 and log2-FC = < 0.5 or > 1) between the NAT versus TN group (Fig. 2b). The distribution of DALs across both ionization modes in both sample groups is shown using volcano plots (Fig. 2c). Out of a total of 183 DAL features in tissue samples, 144 (79%) showed higher abundance in the NAT than the TN group. In contrast, 85 of 132 (65%) features in serum samples showed lower abundance in the NAT than the TN group (Fig. 2c).

**Fig. 3:**
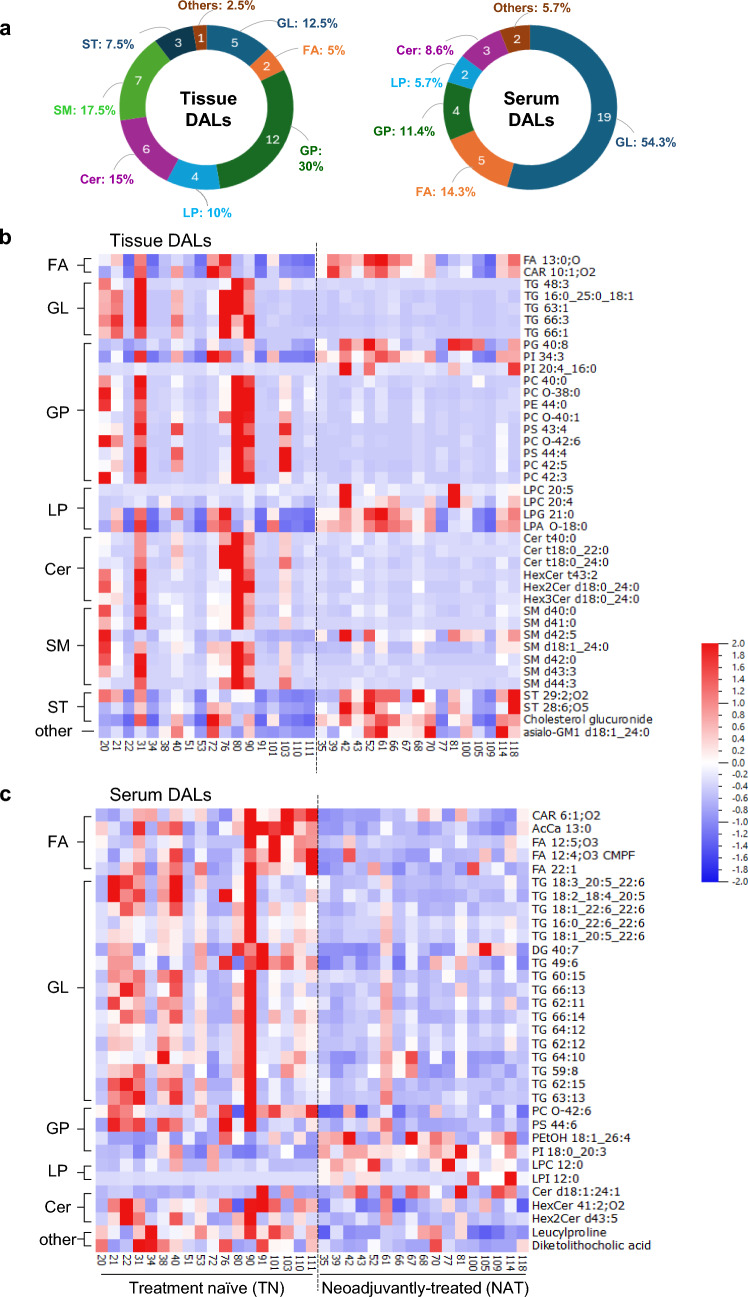
Differentially abundant lipid (DAL) metabolites in the NAT versus TN group. (**a**) Pie charts showing distribution of different lipid classes that constitute the tissue and serum DALs. (**b, c**) Heatmaps showing the pattern of abundance of DALs between treatment groups in (**b**) tissue and in (**c**) serum samples. Cer, ceramide; FA, fatty acids/acyls; GL, glycerolipids; GP, glycerophospholipids; LP, lysophospholipids; NAT, neoadjuvant FOLFIRINOX-treated; SM, sphingomyelins; ST, sterol lipids; TN, treatment naïve

Next, lipid features were annotated according to the recommendations of the Lipidomics Standards Initiative (LSI) (Fahy et al. 2009; Fahy et al. 2005). Comparison of MS profiles with the in-house lipid library, LipidSearch (lipid annotation software) and the public database LIPID MAPS allowed identification of DALs, which amounted to a total of 40 tissue and 35 serum DALs (Fig. 2d). Following annotation, only lipid features with confidence levels 1–3 were selected, where level 1 indicates confirmation with an authentic standard, level 2 indicates putative identification based on MS/MS fragmentation and database match, and level 3 indicates class-level identification. The complete list of DALs for tissue and serum are provided in Table 2 and Table 3, respectively, and associated raw data in Supplementary Data file. All these features have a PQC RSD < 30% and none were excluded following the blank exclusion criteria (Fig. S2b). In this study, two serum DALs were confirmed as Level 1 using in-house reference compounds (matching retention time and MS/MS spectra). All other DALs were Level 2 or 3, based on MS/MS fragmentation and database/library matching (e.g., LipidSearch). A total of 22 out of 40 tissue DALs (Table 2) and all 35 serum DALs (Table 3) were statistically significant even after correction for FDR by the Benjamini–Hochberg procedure. Among tissue DALs (Table 2), 16 belonged to the lipid class glycerophospholipids (GP), followed by 14 sphingolipids [SP: 6 ceramides (Cer), 7 sphingomyelins (SM), and 1 glycosphingolipid], 5 glycerolipids (GL), 3 sterols (ST), and 2 fatty acids/acyls (FA). Interestingly, 19 out of 35 serum DALs belonged to GL, followed by 6 GP, 5 FA, and 3 SP-Cer, while none were SM (Table 3**, **Fig. 3a). MS also detected two differentially abundant non-lipid features in serum samples: both leucylproline and diketolithocholic acid were significantly lower in the NAT versus TN group (Table 3).

**Table 2:** List of differentially abundant lipid (DAL) metabolites in the TN versus NAT tissue samples

Class	Metabolite name	Level of confidence	FC	p-value	Adj. p-value
**Tissue_POS**					
FA (fatty acid/acyl)	FA 13:0;O	3	− 1.18	0.04*	0.06
	CAR 10:1;O2	3	− 1.11	0.04*	0.06
GL (glycerolipid)	TG 48:3	3	0.89	0.02*	0.04*
	TG 16:0_25:0_18:1	2	0.87	0.02*	0.05*
	TG 63:1	3	0.45	0.009**	0.09
	TG 66:3	3	0.94	0.03*	0.06
	TG 66:1	3	1.62	0.01*	0.04*
GP (glycerophospholipid)	PG 40:8	3	− 1.24	0.004**	0.06
	PI 34:3	3	− 1.88	0.04*	0.06
	PI 20:4_16:0	2	− 1.97	0.007**	0.04*
	LPC 20:5	2	− 1.23	0.05*	0.04*
	LPC 20:4	2	− 1.08	0.02*	0.06
	LPG 21:0	3	−0.99	0.04*	0.07
SP-Cer (sphingolipid-ceramide)	Cer t40:0	3	0.45	0.04*	0.09
	Cer t18:0_22:0	3	0.77	0.03*	0.10
	Cer t18:0_24:0	2	1.19	0.04*	0.06
	HexCer t43:2	3	2.33	0.01*	0.04*
	Hex2Cer d18:0_24:0	3	1.92	0.04*	0.06
	Hex3Cer d18:0_24:0	2	2.01	0.02*	0.05*
SP-SM (sphingolipid- sphingomyelin)	SM d40:0	2	0.94	0.03*	0.10
	SM d41:0	2	3.83	0.004**	0.04*
	SM d42:5	2	− 0.63	0.03*	0.06
	SM d18:1_24:0	2	− 0.05	0.03*	0.05*
	SM d42:0	2	5.61	0.005**	0.04*
	SM d43:3	3	1.73	0.03*	0.04*
	SM d44:3	3	3.48	0.03*	0.23
SP-Gsl (sphingolipid-glycosphingolipid)	asialo-GM1 d18:1_24:0	2	− 1.85	0.03*	0.06
ST (sterol lipid)	ST 29:2;O2	3	− 0.99	0.04*	0.06
	ST 28:6;O5	3	− 0.91	0.01*	0.04*
	Cholesterol glucuronide	3	− 1.58	0.03*	0.06
**Tissue_NEG**					
GP (glycerophospholipid)	PC 40:0	2	2.11	0.04*	0.04*
	PC O-38:0	3	1.93	0.008**	0.03*
	PC O-40:1	2	3.50	0.01*	0.03*
	PC O-42:6	3	1.29	0.02*	0.03*
	PC 42:5	3	1.64	0.02*	0.03*
	PC 42:3	3	1.78	0.01*	0.03*
	PE 44:0	2	2.48	0.04*	0.04*
	PS 43:4	3	1.18	0.01*	0.03*
	PS 44:4	3	1.09	0.01*	0.03*
	LPA O-18:0	3	− 1.66	0.03*	0.04*

DAL species identified individually for both positive (POS) and negative (NEG) ionization modes were classified according to LIPID MAPS. Data presented including fold-change (FC) and *P*-values were calculated comparing treatment-naïve (TN) versus neoadjuvantly treated (NAT) samples. Adjusted p-values account for the Benjamini–Hochberg correction procedure for false discovery. For both p- and adj. p-values, #*p* < .1, *p < .05, **p < .01 between TN and NAT samples. CAR/AcCa, acylcarnitine; HexCer, hexosylceramide; Hex2Cer, dihexosylceramide; Hex3Cer, trihexosylceramide; LPA, lysophosphatidic acid; LPC, lysophosphatidylcholine; LPG, lysophosphatidylglycerol; PC, phosphatidylcholine; PE, phosphatidylethanolamine; PG, phosphatidylglycerol; PI, phosphatidylinositol; PS, phosphatidylserine; TG, triacylglycerol

**Table 3:** List of differentially abundant lipid (DAL) metabolites in the TN versus NAT serum samples

Class	Metabolite name	Level of confidence	FC	*p*-value	Adj. *p*-value
**Serum_POS**					
FA (fatty acid/acyl)	FA 12:5;O3	3	2.23	0.04*	0.04*
	AcCa 13:0	2	1.95	0.0002**	0.003**
	CAR 6:1;O2	3	1.27	0.004**	0.01*
GL (glycerolipid)	TG 18:3_20:5_22:6	2	1.45	0.03*	0.04*
	TG 18:3_20:5_22:6	2	1.41	0.01*	0.02*
	TG 18:2_18:4_20:5	2	2.06	0.002**	0.01*
	TG 18:1_22:6_22:6	2	1.47	0.01*	0.01*
	TG 16:0_22:6_22:6	2	1.07	0.02*	0.03*
	TG 18:1_20:5_22:6	2	1.50	0.002**	0.01*
	TG 49:6	3	1.43	0.003**	0.01*
	TG 60:15	3	1.06	0.03*	0.03*
	TG 66:13	3	1.99	0.02*	0.03*
	TG 62:11	3	1.16	0.04*	0.04*
	TG 66:14	3	2.12	0.003**	0.01*
	TG 64:12	3	1.65	0.004**	0.01*
	TG 62:12	3	1.69	0.007**	0.02*
	TG 62:12	3	1.31	0.01*	0.02*
	TG 64:10	3	1.17	0.003**	0.01*
	TG 59:8	3	1.10	0.01*	0.02*
	TG 62:15	3	1.24	0.02*	0.03*
	TG 63:13	3	1.37	0.01*	0.02*
	DG 40:7	3	1.63	0.02*	0.03*
GP (glycerophospholipid)	PC O-42:6	3	1.14	0.003**	0.01*
	PS 44:6	3	1.05	0.02*	0.03*
	LPC 12:0	2	− 1.80	0.01*	0.02*
	LPI 12:0	3	− 2.16	0.002**	0.01*
SP-Cer (sphingolipid-ceramide)	HexCer 41:2;O2	2	1.01	0.008**	0.02*
	Hex2Cer d43:5	3	1.59	0.05*	0.04*
–	Leucylproline	3	0.82	0.0002**	0.003**
**Serum_NEG**					
FA	FA 12:4;O3 (CMPF)	1	1.45	0.04*	0.04*
	FA 22:1	1	1.28	0.005**	0.02*
GP	PI 18:0_20:3	2	− 0.86	0.00005**	0.0003**
	PEtOH 18:1_26:4	2	− 2.04	0.04*	0.04*
SP-Cer	Cer d18:1:24:1	3	− 1.79	0.04*	0.04*
–	Diketolithocholic acid	3	1.29	0.02*	0.04*

DAL species identified individually for both positive (POS) and negative (NEG) ionization modes were classified according to LIPID MAPS. Data presented including fold-change (FC) and *P*-values were calculated comparing treatment-naïve (TN) versus neoadjuvantly treated (NAT) samples. Adjusted p-values account for the Benjamini–Hochberg correction procedure for false discovery. For both p- and adj. p-values, #*p* < .1, *p < .05, **p < .01 between TN and NAT samples. CAR/AcCa, acylcarnitine; DG; diacylglycerol; HexCer, hexosylceramide; Hex2Cer, dihexosylceramide; LPC, lysophosphatidylcholine; LPI, lysophosphatidylinositol; PC, phosphatidylcholine; PEtOH, phosphatidylethanol; PI, phosphatidylinositol; PS, phosphatidylserine; TG, triacylglycerol

Abundance of DALs across samples from both tissue and serum are presented using heatmaps (Fig. 3b, c). Except for serum diacylglycerol DG 40:7, all lipids in class GL in both sample groups were triacylglycerols (TG) and interestingly, the abundance of all GL was significantly lower in the NAT versus TN group. The pattern of FA abundance in the NAT versus TN group differed between both sample groups: the abundance after NAT was significantly lower for all five serum FA, while it was higher for both tissue FA. Regarding GP lipids, all lysophospholipids (LP) and phosphatidylinositols (PI) were higher, whereas all phosphatidylcholines (PC) and phosphatidylserines (PS) were lower in the NAT versus TN group in both tissue and serum samples. ST, which were only seen in tissue DALs, were all higher in the NAT than the TN group. SP were generally lower in the NAT versus TN group, including all six Cer in tissue and 2 out of 3 in serum, and 5 out of 7 tissue SM (Fig. 3**, **Table 2,3).

### Correlation between levels of DALs and clinical parameters

**Fig. 4:**
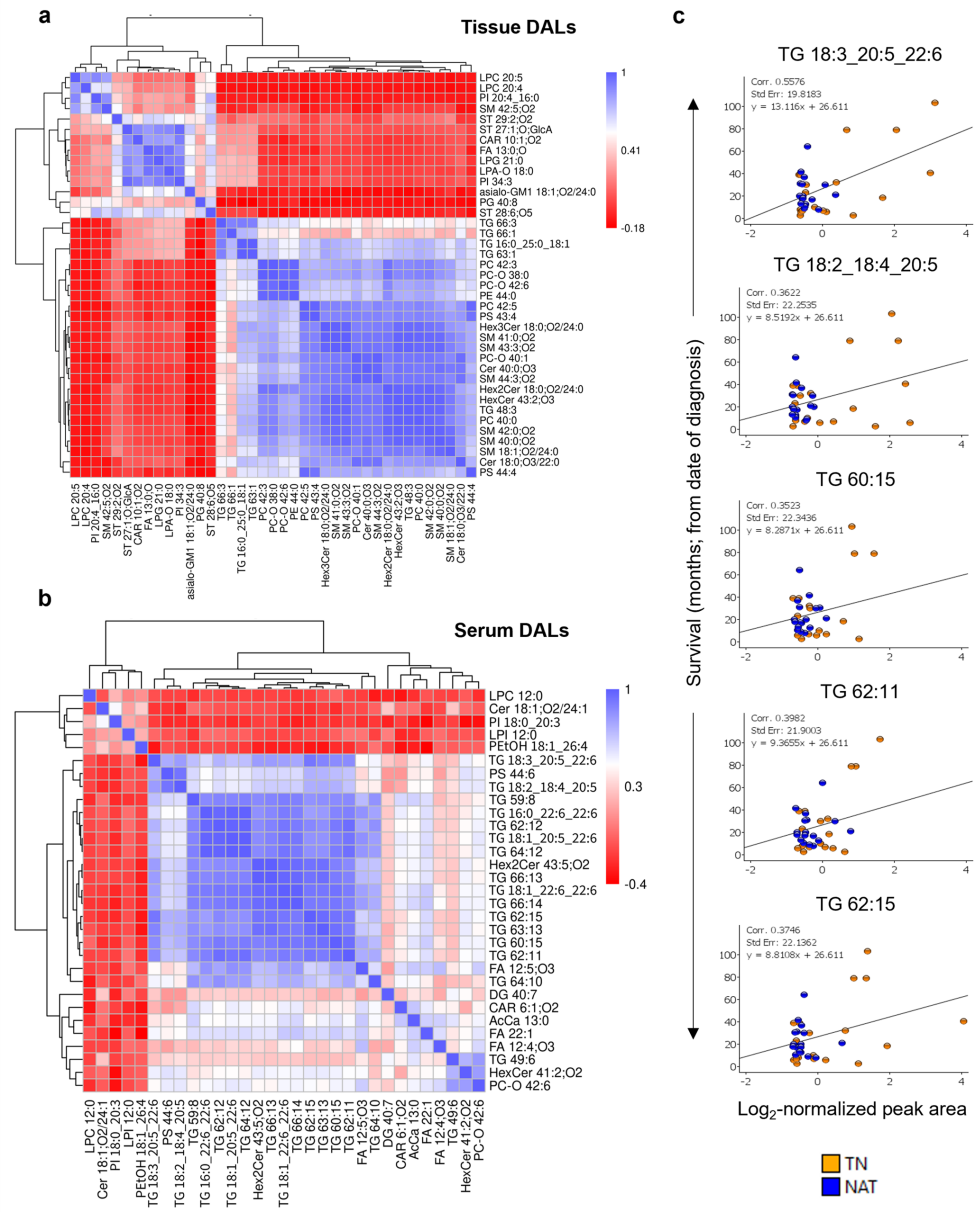
Correlation analysis. (**a, b**) Heatmaps showing correlation between differentially abundant lipids (DALs) in (**a**) tissue and (**b**) serum samples. The strength and direction of correlation is indicated with values 1 (blue), −1 (red) and 0 (white) represent perfect positive, perfect negative, and no correlation, respectively. (**c**) Scatter plots showing correlation between the abundance of serum DALs and survival calculated from the date of diagnosis. Cer, ceramides; HexCer, hexosylceramides; FA, fatty acids/acyls; GL, glycerolipids; GP, glycerophospholipids ‒ PC, PE, PG, PI, PS, PEtOH; LP, lysophospholipids ‒ LPA, LPC, LPG; NAT, neoadjuvant FOLFIRINOX-treated; SM, sphingomyelins; ST, sterol lipids; TG, triacylglycerols; TN, treatment naïve

Correlation analysis between various individual DALs revealed several tissue DALs with strong correlation (including 5 TG, 6 PC, 6 SM, 2PS, and 5 SP-Cer; Fig. 4a) while for serum DALs, 14 TG, one PS and one Hex2Cer were found to correlate (Fig. 4b). When combined for all samples in both treatment groups, none of the serum or tissue DALs showed strong correlation with the clinicopathological parameters (Table 1), including age, BMI, tumour size, OS, and serum markers. Five serum DALs showed a moderate positive correlation (Fig. 4c), while none of the tissue DALs correlated with OS for the entire cohort. Interestingly, all five DALs were TG containing long chained poly unsaturated fatty acid side chains (TG 18:2_18:4_20:5, TG 18:3_20:5_22:6, TG 60:15, TG 62:11, and TG 62:15; Fig. 4c). Fragmentation spectra for these lipids are provided in Fig. S3. Next, the correlation between DALs and OS was assessed separately for each treatment group, which identified 6 tissue and 4 serum DALs for the NAT group and 1 tissue and 9 serum DALs for the TN group (Fig. S4). However, as a significant proportion of the patients in the study cohort (9 of 35) were still alive at last follow-up, Cox regression analysis was performed, which showed considerable overlap in survival between NAT and TN groups (Fig S5).

### Correlation between levels of DALs and change in serum CA 19–9 following NAT

**Fig. 5:**
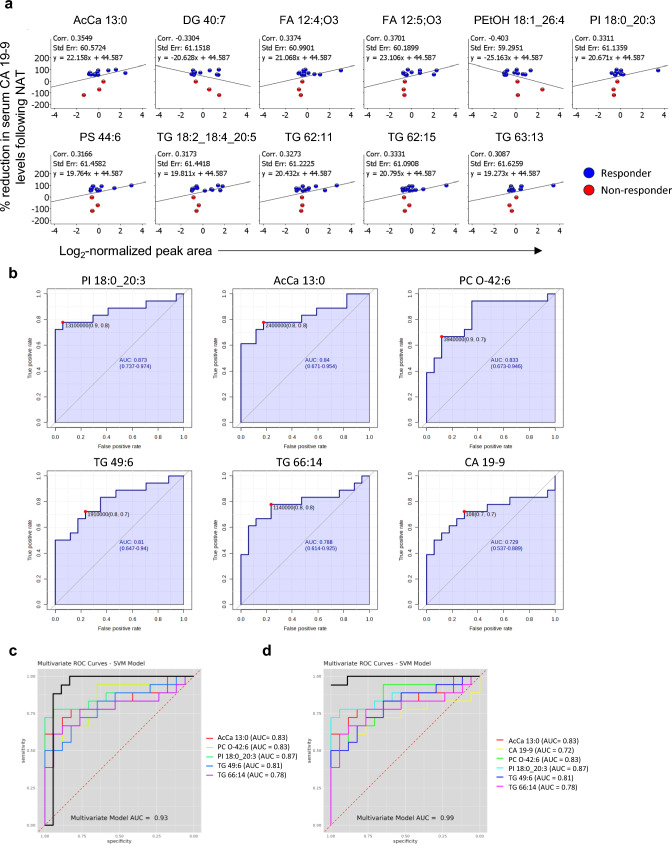
Lipid metabolites to distinguish between NAT and TN groups. (**a**) Scatter plots showing correlation between the abundance serum DALs on X-axis and percentage reduction in serum CA 19–9 following NAT on Y-axis. (**b**) Individual ROC curves generated using MetaboAnalyst for five serum DALs with highest AUC and CA 19–9. (**c, d**) Multivariate ROC curves computed using LipidOne for (**c**) five serum DALs alone, and (**d**) combined with CA 19–9. AcCa, acylcarnitine; AUC, area under curve; CA 19–9, carbohydrate 19–9 antigen; Cer, ceramides; DAL, differentially abundant lipid; HexCer, hexosylceramides; LPI, lysophosphatidylinositols; NAT, neoadjuvant FOLFIRINOX-treated; PC, phosphatidylcholines; PI, phosphatidylinositols; PS, phosphatidylserines; ROC, receiver operating characteristic; SVM, support vector machine; TG, triacylglycerols; TN, treatment naïve

To identify potential biomarkers for NAT response in PDAC, the correlation between abundance of both tissue and serum DALs and the change in CA 19–9 level between pretreatment and preoperative blood samples for patients in the NAT group (n = 17) was analysed. Pretreatment serum CA 19–9 data was accessible for 15 NAT patients. Following NAT, the level of serum CA 19–9 was reduced > 50% in 12 patients (responders) but remained unchanged or increased compared to pretreatment levels in the remaining three patients (non-responders; Table S1). Except for cholesterol glucuronide (r = 0.71), there was no strong correlation [r = +/− (0.7 to 1)] between the abundance of any of the serum or tissue DALs and % reduction in serum CA 19–9 following NAT. Several DALs showed a moderate correlation [r = +/− (0.3 to 0.7)]: 11 out of 35 serum DALs (Fig. 5a) and 26 out of 40 tissue DALs (Fig. S6). The most dominant lipid classes for these DALs were TG and phospholipids (GP and LP) for serum and tissue, accounting for 12 and 11 DALs, respectively. The top ()five tissue DALs with the highest r-value of correlation were cholesterol glucuronide, CAR 10:1;O2, ST 29:2;O2, PI 34:3, and LPG 21:0 (Fig. S6). Next, correlation between serum DALs and reduction in CA 19–9 was assessed following exclusion of the three non-responders. It identified 20 out of 35 serum DALs showing moderate correlation with the reduction in CA 19–9 following NAT (Fig. S7). Interestingly, the majority of these showed a better r value than when all NAT samples were analysed together (Fig. 5a, Fig. S7). Among these serum DALs, the top five with the highest r value were TG 66:13, Hex2Cer d43:5, TG 18:1_22:6_22:6, TG 66:14, and TG 59:8 (Fig.S7).

### Lipidomic signatures distinguishing NAT from TN PDAC

The performance of all serum DALs to distinguish post-NAT from TN samples was first evaluated by univariate ROC analysis. Of a total of 32 DALs, 23 showed a significant but moderate discriminatory potential with the area under ROC curves (AUC) in the range of 0.66 to 0.87 and *p* < .05 (Table S2). These DALs mainly belonged to the lipid classes GL (Qin et al. 2020) followed by FA (Gugenheim et al. 2022), GP (Springfeld et al. 2023) and SP (Park et al. 2021). The individual ROC curves for the top five DALs with the highest AUC (0.78 to 0.87;*p* < .05) are presented in Fig. 5b. These included PI 18:0_20:3, AcCa 13:0, PC O-42:6, TG 49:6, and TG 66:14. Fragmentation spectra of the former three (level 2 lipids) are provided in Fig. S8. Four of these except TG 49:6 also showed moderate positive correlation with the reduction of serum CA 19–9 following NAT (Fig. 5a). Interestingly, the individual performance of a total of 15 serum DALs was better than that of CA 19–9 (the latter having AUC = 0.72 and 95% CI = 0.53–0.89) but the difference in performance was not statistically significant (Table S2). Patients in the NAT versus TN groups differed significantly in terms of age [AUC = 0.71, 95% CI = 0.49–0.86] and the presence of comorb()idities [AUC = 0.68, 95% CI = 0.53–0.82]. Both clinical features displayed a slightly lower AUC than CA 19–9 but were statistically significant (*p* < .05; Table S2, Fig. S9). Next, a multivariate exploratory ROC analysis was performed to improve the prediction. The top five DALs with the highest discriminatory ability (represented by AUC) to distinguish NAT from TN groups, performed better when combined rather than individually (AUC = 0.93 and 95% CI = 0.79‒1; Fig. 5c). Interestingly, this performance was further improved when combined with CA 19–9 (AUC = 0.99 and 95% CI = 0.81‒1; Fig. 5d), which remained unaffected by the differences in age, comorbidities, BMI or gender between the two groups (Fig. S9).

## Discussion

Neoadjuvant chemotherapy has become the standard of care for locally advanced PDAC, but exploiting its full potential is hampered by limited insight into its molecular impact on the tumour and by the lack of biomarkers of treatment response both during and after completion of therapy and/or survival (Springfeld et al. 2023; Gugenheim et al. 2022; Oba et al. 2020; van Dam et al. 2022). Thus, there is an urgent need to further characterize the effects of NAT in PDAC. Several recent studies suggest that advanced multi-omics techniques may have great potential in this respect (Amrutkar et al. 2023; Kaoutari et al. 2021; Stillger et al. 2024; Nemkov et al. 2024). A recent multi-omics analysis of PDAC specimens revealed NAT-induced immune and metabolic alterations in PDAC (Amrutkar et al. 2023). Our proteome profiling study revealed the impact of NAT on tumour and systemic metabolism with a markedly lower expression of metabolism-related proteins in NAT versus TN PDACs (Amrutkar et al. 2025). To further investigate the impact of NAT on metabolism, we recently conducted global metabolomic profiling of tumour tissue and matched serum samples from neoadjuvant FOLFIRINOX-treated and TN PDACs (Fu et al. 2021). That study showed differential abundance of several metabolites, NAT-induced changes in amino acid and nucleotide metabolic pathways, and their correlation with reduced CA 19–9 following NAT. However, due to methodological limitations, our metabolomic profiling detected mainly polar, water-soluble metabolites, while it failed to identify most hydrophobic metabolites such as lipids (Wang et al. 2020; Belhaj et al. 2021). Lipids are important biomolecules involved in diverse physiological processes. In cancer, lipids play a multifaceted role by supplying energy and biomass for membrane formation, and by acting as signalling molecules that influence the lipid metabolism and tumour microenvironment (Yin et al. 2022; Wu et al. 2024). In line with this, tumour tissue and blood lipid profiles have been increasingly investigated in search of potential prognostic markers of PDAC (Wolrab et al. 2022; Peterka et al. 2024; Horejsi et al. 2023). Hence, we considered it important to investigate the impact of NAT on PDAC lipid metabolite profiles in the same cohort as for the global metabolomics study (Amrutkar et al. 2025). This was achieved by LC–MS-based lipidomic profiling using a hypothesis-generating global analysis approach, which discovers all detectable lipids in a biological sample, including unknown lipids (Han 2016; Zullig et al. 2020).

Lipidomic profiling identified nearly 11,000 and 24,000 lipid features in tissue and serum samples, respectively, but the abundance of only 183 (1.7%) and 132 (0.6%) of these was significantly altered between both treatment groups. Further analysis identified a total of 40 unique DALs in PDAC tissue and 35 in serum samples. Notably, more than two-thirds of both differential features and DALs were less abundant in tissue and serum samples from NAT versus TN PDACs. This possibly indicates NAT-induced metabolic reprogramming or slowdown, as we and others have previously reported (Amrutkar et al. 2023; Amrutkar et al. 2025; Tang et al. 2023, Teng et al. 2024). Although MS mapped a vast number of lipid features in both tissue and serum samples, the number of features that were significantly altered between the treatment groups was much lower, only around 1%. This is likely due to the significant metabolic heterogeneity, a hallmark of PDAC that exists between samples in each of the individual treatment groups (Qin et al. 2020; De Santis et al. 2024).

The distribution of DALs according to the lipid classes differed between tissue and serum samples (Fahy et al. 2009). Glycerophospholipids (GP) and sphingolipids (SP) accounted for 30 of the 40 tissue DALs, while the remaining 10 DALs were glycerolipids (GL), sterol lipids (ST) and fatty acyls (FA). In contrast, 19 of 35 serum DALs were GL, whereas GP, FA, and SP accounted for the remaining 16 DALs. GP and SP were the most abundant lipid classes in tissue DALs, and both are fundamental components of biological membranes. GP, a class of phospholipids characterized by a glycerol backbone, are involved in membrane structure, stability, and function, as well as in cell signalling and protein anchoring (Fahy et al. 2005). Among GP, phosphatidylcholines and phosphatidylserines were less abundant, while phosphatidylinositols and lysophospholipids were more abundant in NAT than TN, in both tissue and serum samples. SP are characterized by a sphingosine backbone and are known to be involved in a wide range of cellular functions, including growth regulation, cell migration, and apoptosis (Fahy et al. 2005). All ceramides ‒ a type of SP ‒ were lower in NAT versus TN in both sample groups, except serum Cer d18:1:24:1. Ceramides are suggested to play diverse roles in cancer, including apoptosis, proliferation, metabolism and treatment resistance (Wajapeyee et al. 2024; Zhakupova et al. 2025). Sphingomyelins, another type of SP, showed a mixed pattern, while ST were higher in the NAT than TN group in tissue samples. Of note, none of the serum DALs belonged to either SP or ST classes.

GL, the most abundant of phospholipids characterized by a glycerol backbone esterified with fatty acids, are essential components of cellular membranes and play role in energy storage and signalling (Fahy et al. 2005; Fu et al. 2021). Notably, all serum GL except DG 40:7 and all tissue GL were TG, which were less abundant in the NAT versus the TN group. TG are primary dietary lipids that serve as major energy storage molecules. Although studies on the relation between serum TG and PDAC remain sparse, elevated serum TG levels are a known component of the metabolic syndrome, which has been linked to an increased risk of PDAC (Miyashita et al. 2024; Song et al. 2024). Like GL, FA showed low abundance in NAT versus TN in serum samples, however, the opposite pattern was seen in tissue samples. FA are the building blocks of many complex lipids, including phospholipids, SP and TG, and their metabolism is reprogrammed to support cancer growth (Koundouros and Poulogiannis 2020; Rohrig and Schulze 2016). FA in cancer cells, including those of PDAC, derives from de novo synthesis, and increased FA synthesis has been linked to PDAC progression and poor survival, as well as gemcitabine resistance (Koundouros and Poulogiannis 2020; Li et al. 2023; Tadros et al. 2017).

In search of potential markers of treatment response, we first investigated the correlation between lipid metabolite abundance and change in serum CA 19–9 in the NAT group alone. CA 19–9 was not reduced in all patients following NAT, as it remained unchanged or increased compared to pretreatment levels in three patients (non-responders). For patients with lower CA 19–9 levels following NAT (responders), the % reduction in CA 19–9 correlated moderately positively with 11 serum and 26 tissue DALs, most of which belonged to the lipid classes TG for serum and phospholipids for tumour tissue. Second, we investigated the potential of all serum DALs along with CA 19–9 to distinguish NAT from TN samples. Interestingly, 15 serum DALs individually performed better than CA 19–9 alone, and five of these with the best performance (PI 18:0_20:3, AcCa 13:0, PC O-42:6, TG 49:6, and TG 66:14) were selected for further analysis. Of all combinations tested, a model consisting of these five serum DALs together with CA 19–9 showed the best discriminatory ability (AUC = 0.99) to distinguish NAT from TN samples, which was not affected by two potential confounders – age and comorbidity – that could possibly influence lipid profiles independently of treatment effect. Notably, the abundance of these serum DALs, except for TG 49:6, correlated moderately positively with the % reduction of serum CA 19–9 following NAT. Response to neoadjuvant treatment in PDAC is currently based on preoperative assessment of the change in serum CA 19–9 as well as tumour imaging and postoperative histological grading of tumour regression (TRG) (Springfeld et al. 2023; Gugenheim et al. 2022; Holm et al. 2024). As the latter is done postoperatively, the TRG results cannot be used to guide decision regarding a possible alteration of the NAT regimen. Moreover, TRG suffers from marked interobserver variation (Holm et al. 2024; Janssen et al. 2022). In contrast, serum CA 19–9 allows rapid and non-invasive assessment of treatment at multiple timepoints during the course of NAT. Hence, change in serum CA19-9 was used as a surrogate for treatment response and correlated with the identified DALs (in isolation or in combination with CA 19–9), despite the limited sensitivity and specificity of CA 19–9. Taken together, the abundance of several lipid metabolites was found altered following NAT in both tissue and serum samples, indicating the importance of investigating lipid metabolism in PDAC. Increasing evidence shows that dysregulated lipid metabolism represents a key metabolic alteration in human cancers, including PDAC, that is relevant to disease progression as well as treatment resistance (Yin et al. 2022; Fu et al. 2021; Zhang et al. 2025). The present study has three main findings. First, the lipid species showing the most prominent changes in abundance between the NAT and TN groups belonged to GP, SP, GL (mainly TG), and FA. The former two were dominant in tissues, the latter was dominant in serum samples. These four lipid classes have previously been reported to play a role in tumour progression (Peterka et al. 2024; Zhakupova et al. 2025; Song et al. 2024; Koundouros and Poulogiannis 2020). Second, the lesser abundance of various lipid metabolites following neoadjuvant FOLFIRINOX in both tissue and serum samples, as observed in this study, might indicate reduced metabolic activity in PDAC patients following NAT but could also be associated with treatment-induced hepatic stress, loss of adipose-derived lipids or systemic inflammation. Third, this study proposes a potential biomarker panel of serum CA 19–9 combined with five serum DALs (PI 18:0_20:3, AcCa 13:0, PC O-42:6, TG 49:6, and TG 66:14) to assess the response to NAT in PDAC.

This study is the first to reveal the potential of lipidomics to investigate metabolic alterations in PDAC following neoadjuvant FOLFIRINOX treatment. However, this approach has several limitations. First, the sample groups show inevitably a different composition in terms of resectability status, age and comorbidity. Moreover, given the lack of pretreatment samples for the NAT group, TN PDACs were used as a proxy control, which also weakens the strength of the comparison. Second, lipid profiles are increasingly suggested to be influenced by several factors such as diet, physical activity, concurrent medications, and the time of sampling relative to treatment (Padro et al. 2023; Herink 2000; Bogusiewicz et al. 2024; Wang and Xu 2017). However, these factors could not be considered due to the lack of relevant data. Third, the strength of DALs as potential biomarkers to assess NAT response in PDAC is limited by the relatively small sample size and lack of an external validation cohort in this proof-of-concept study. Therefore, further validation of these findings using a large, independent cohort of samples collected prior to, during and after the treatment, from the same patients is needed. Lastly, intratumour heterogeneity may influence the results from tissue samples, as these were collected randomly from the tumour bed of each PDAC, while the serum results may reflect global metabolism. Despite these limitations, the study demonstrates the potential of lipidomic profiling in discovering biomarkers of NAT effect in PDAC. The study also provides further evidence of altered metabolism following exposure to NAT in PDAC and highlights the potential of serum lipids as prognostic biomarkers. In particular, serum lipid metabolites could potentially be used as biomarkers for early assessment of response to NAT, enabling early adaptation of the treatment regimen (chemotherapeutic switch) should the ongoing treatment be ineffective.

## Conclusions

This proof-of-concept, lipidomic profiling study revealed differential abundance of various lipid metabolites in both tissue and serum following neoadjuvant FOLFIRINOX treatment compared to treatment-naïve PDACs. In tissue samples, the lipids with the most significant alteration following NAT belonged to glycerophospholipid and sphingolipid classes. In contrast, the most common serum DALs were glycerolipids, mainly triacylglycerols, which were less abundant in NAT versus TN. Abundance of 26 tissue and 11 serum DALs correlated moderately with % reduction in serum CA 19–9 following NAT. A serum lipid metabolite panel of PI 18:0_20:3, AcCa 13:0, PC O-42:6, TG 49:6, and TG 66:14 in combination with CA 19–9 could potentially be used for improved monitoring of treatment response in PDAC patients. Validation of these findings in a large independent cohort is needed.

## Supplementary Information

Below is the link to the electronic supplementary material.

Supplementary file1 (PDF 1530 KB)

Supplementary file2 (TXT 37 KB)

Supplementary file3 (PDF 276 KB)

Supplementary file4 (PDF 144 KB)

## Data Availability

Data for differentially altered lipids is provided within supplementary information, and additional data supporting the findings of the study will be made accessible upon reasonable request to the corresponding author (Manoj Amrutkar).

## Abbreviations

**AcCa**: Acylcarnitine
**AUC**: Area under curve
**CA 19****–****9**: Carbohydrate 19–9 antigen
**Cer**: Ceramides
**DAL**: Differentially abundant lipids
**FA**: Fatty acids/acyls
**FOLFIRINOX**: Leucovorin calcium (folinic acid), fluorouracil, irinotecan hydrochloride and oxaliplatin
**GL**: Glycerolipids
**GP**: Glycerophospholipids
**HexCer**: Hexosylceramides
**LC****–****MS**: Liquid chromatography mass spectrometry
**LP**: Lysophospholipids
**LPC**: Lysophosphatidylcholines
**NAT**: Neoadjuvant treatment
**PC**: Phosphatidylcholines
**PCA**: Principal component analysis
**PDAC**: Pancreatic ductal adenocarcinoma
**PE**: Phosphatidylethanolamines
**PI**: Phosphatidylinositols
**PQC**: Pooled quality control
**PS**: Phosphatidylserines
**ROC**: Receiver operating characteristic
**SM**: Sphingomyelins
**SP**: Sphingolipids
**ST**: Sterol lipids
**TG**: Triacylglycerols
**TN**: Treatment-naïve
