# Supplementary material for: Reduced lipid metabolite abundance in human pancreatic cancer and matched serum samples following neoadjuvant FOLFIRINOX treatment
Source: Metabolomics. 2026 Jan 19;22(1):18. doi: 10.1007/s11306-025-02388-z (PMC12816000; doi:10.1007/s11306-025-02388-z)
Supplement: Supplementary file 1 — Supplementary file1 (PDF 1530 KB) [file 11306_2025_2388_MOESM1_ESM.pdf]

# Figure S1

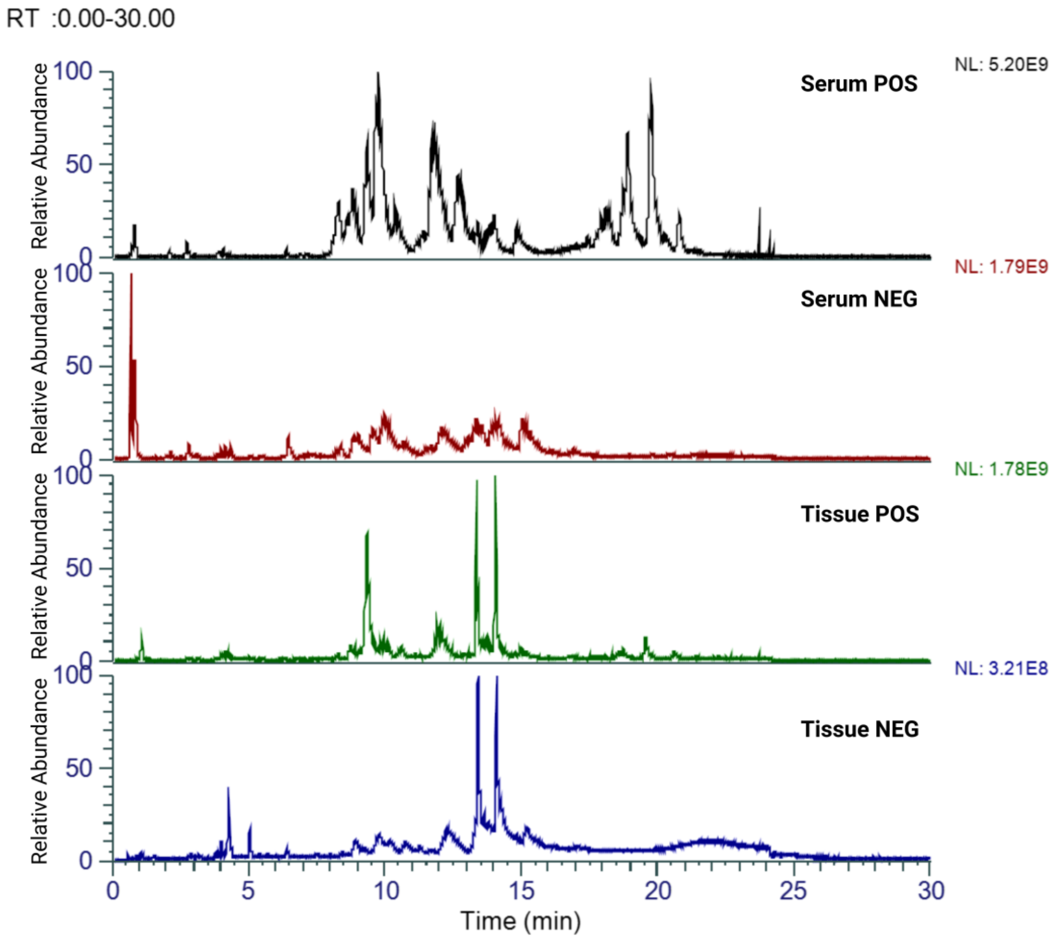


**Fig. S1.** Total ion chromatograms (TIC) of pooled quality control (PQC) samples for serum and tissue for both positive (POS) and negative (NEG) ionization mode. RT, retention time.

**a**

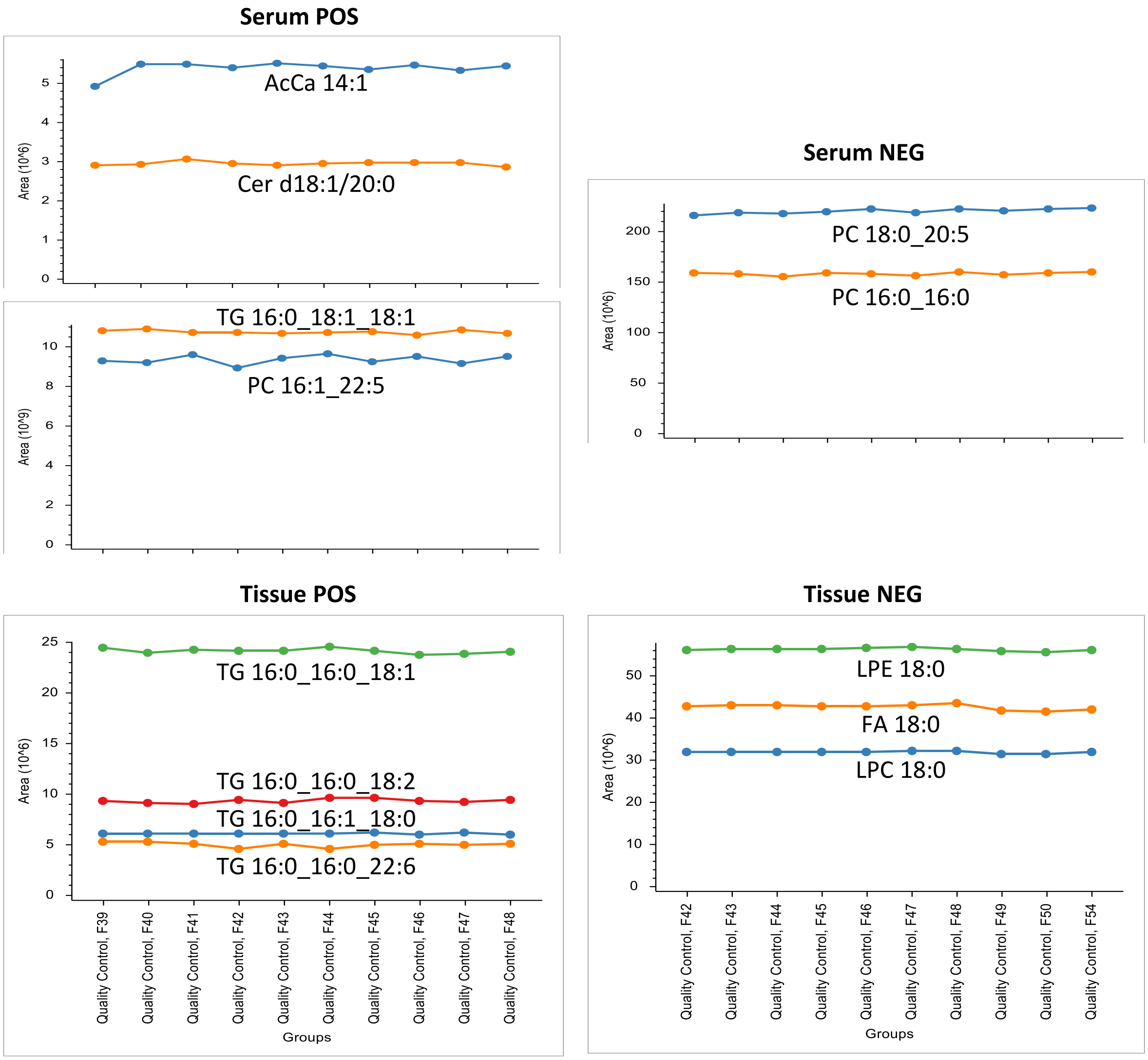


**b**

Relative Standard Deviation (RSD) of metabolite measurements within PQCs

| Category | Tissue POS | Tissue NEG | Serum POS | Serum NEG |
|---|---|---|---|---|
| **Total number of features** | 29180 | 11408 | 37104 | 26708 |
| **Features after filtration** | 7686 | 3246 | 14522 | 9326 |
| **% with PQC RSD <30% of all features detected** | 26.4 | 28.4 | 39.1 | 34.9 |

**Fig. S2. a.** Representative drift plots for one lipid per class, and **b**. RSD proportion of metabolites within pooled quality control (PQC) samples for serum and tissue for both positive (POS) and negative (NEG) ionization mode.

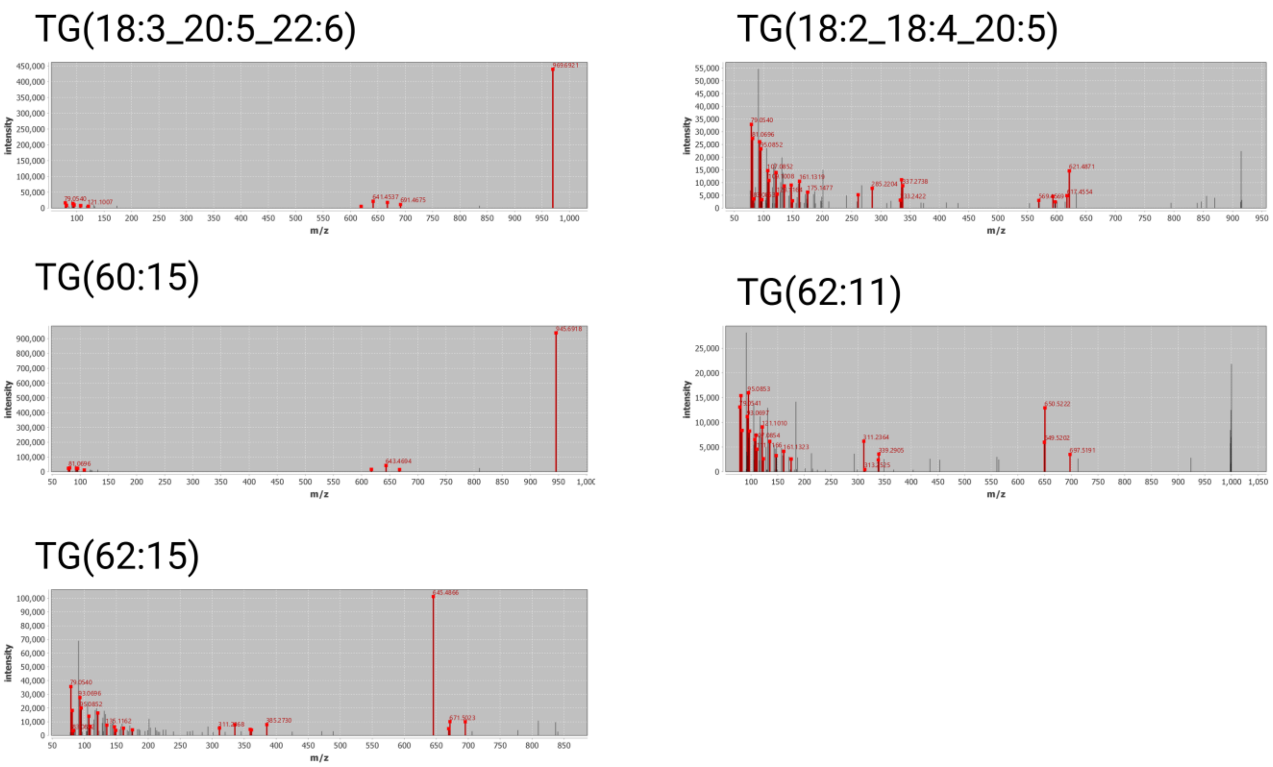


**Fig. S3.** Fragmentation spectra for differentially abundant lipids (DALs) in main Figure 5C. TG, triacylglycerols.

# Figure S4

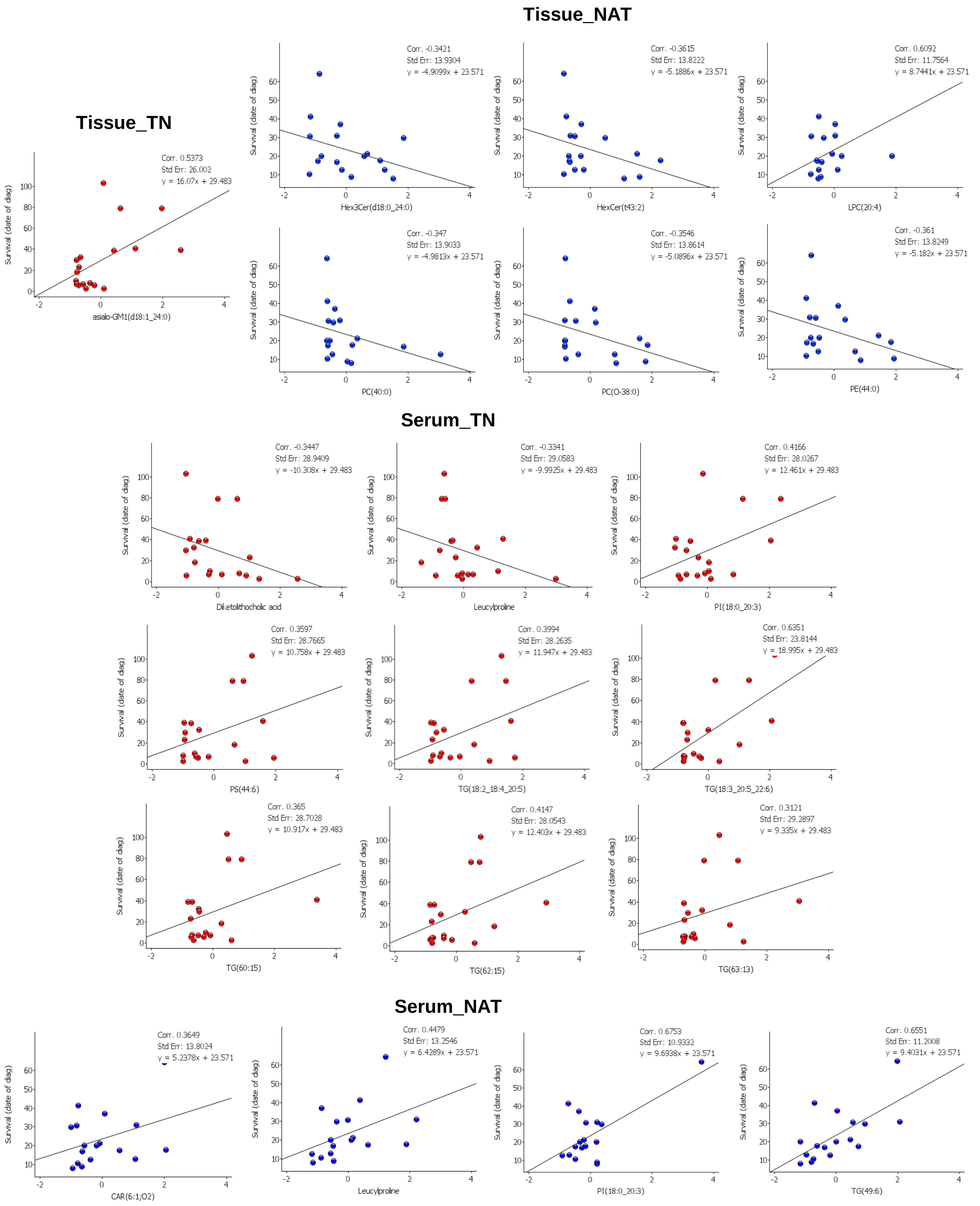


**Fig. S4.** Scatter plots showing correlation between survival and tissue and serum DALs individually for NAT and TN groups. CAR, carnitines; DAL, differentially abundant lipids; HexCer, hexosylceramides; LPC, lysophosphatidylcholines; NAT, neoadjuvant treated; PC, phosphatidylcholines; PE, phosphatidylethanolamines; PI, phosphatidylinositols; PS, phosphatidylserines; TG, triacylglycerols, TN, treatment naïve.

# Figure S5

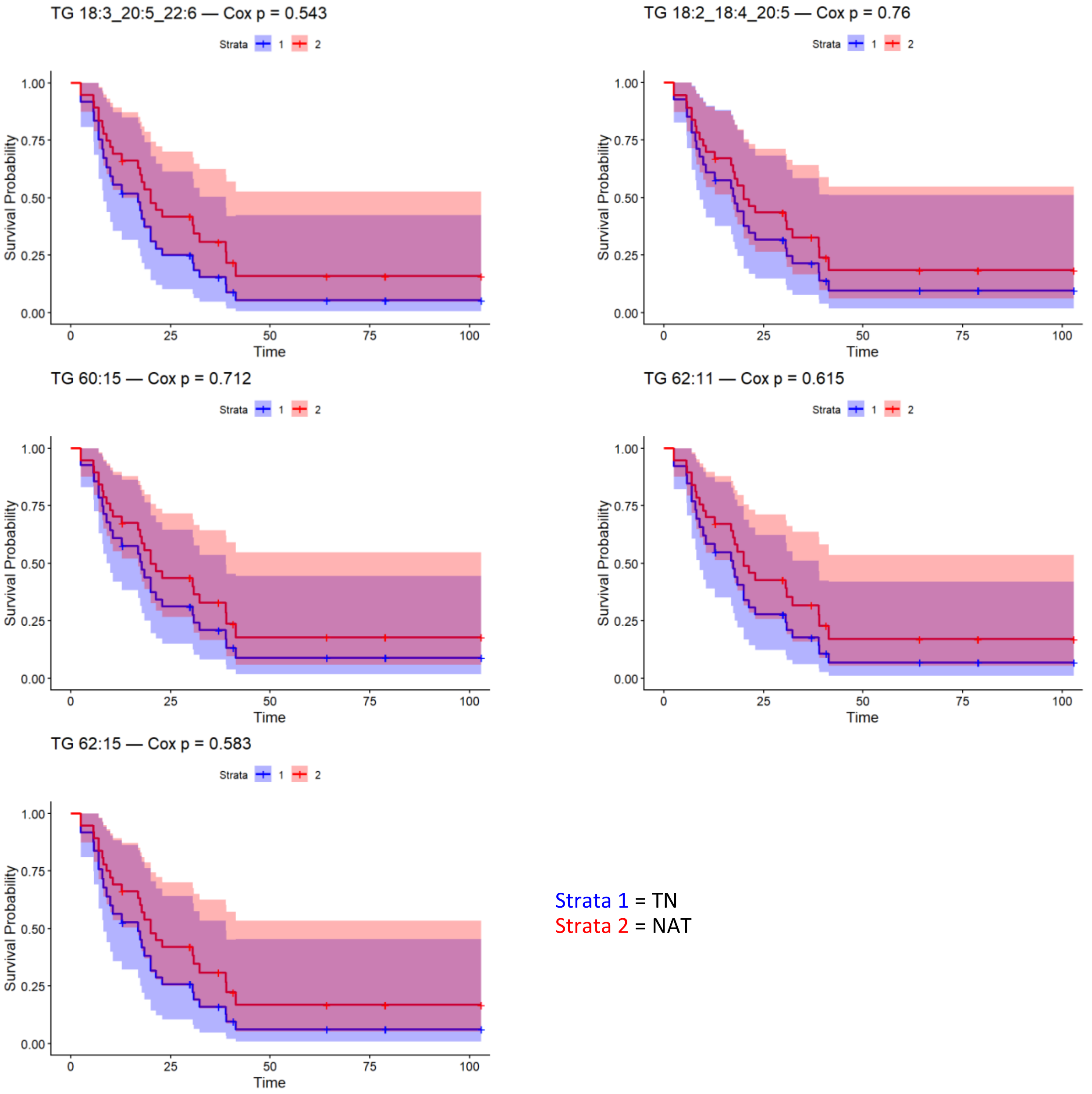


**Fig. S5.** Cox regression plots for serum DALs presented in Fig. 4C, showing correlation between metabolite abundance and survival probability. Survival time (months) on X-axis was calculated from the date of diagnosis. Censored patients were marked with '|' for each treatment group (strata). DAL, differentially abundant lipid; NAT, neoadjuvant FOLFIRINOX-treated; TG, triacylglycerols; TN, treatment naïve**.**

**Figure S6**

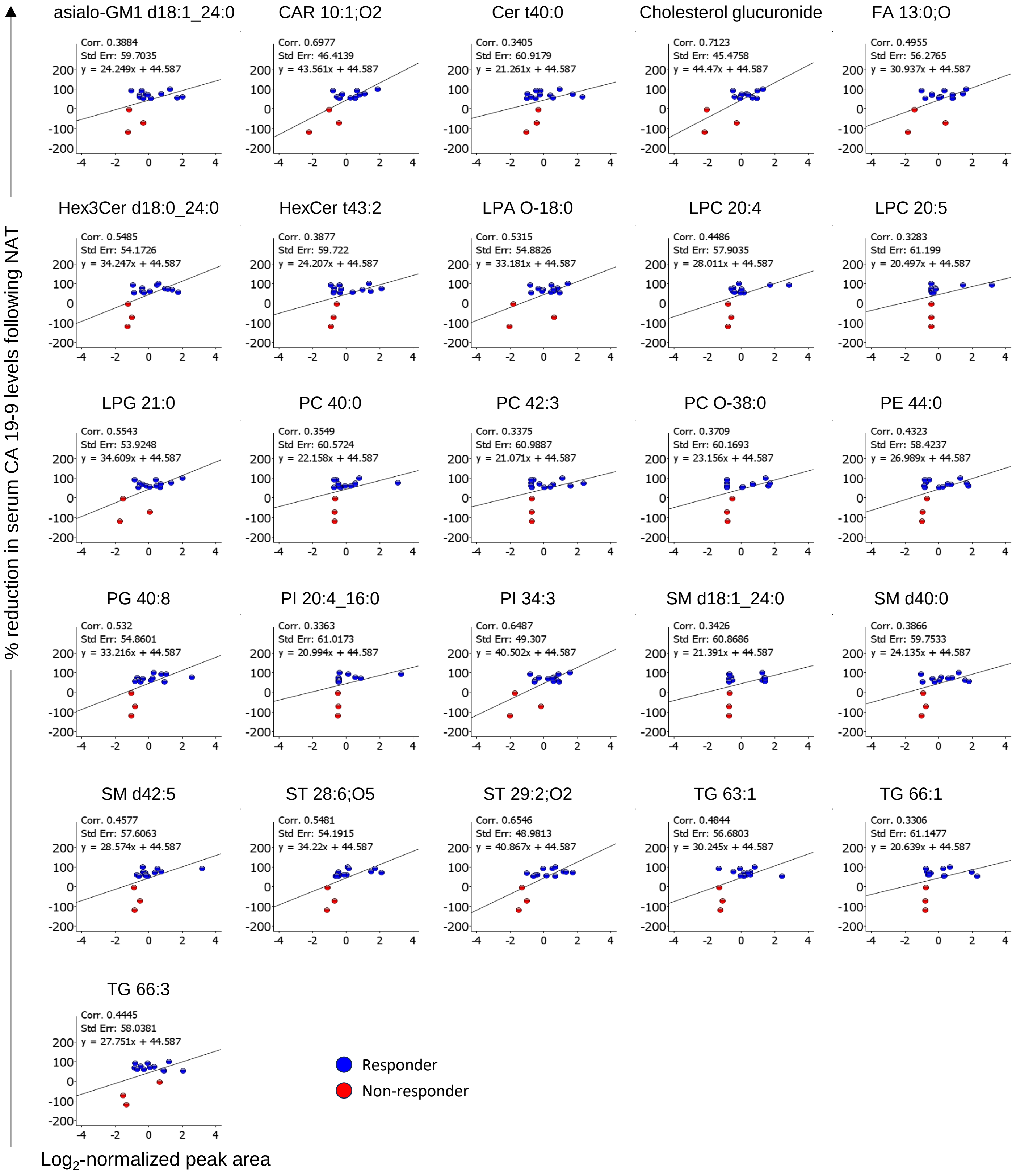


**Fig. S6.** Scatter plots showing correlation between the abundance of tissue DALs (X-axis) and percentage change in serum CA 19-9 level following neoadjuvant FOLFIRINOX treatment (Y-axis). CAR, carnitines; CA 19-9, carbohydrate 19-9 antigen; Cer, ceramides; DAL, differentially abundant lipids; FA, fatty acids/acyls; HexCer, hexosylceramides; LPC, lysophosphatidylcholines; LPG, lysophosphatidylglycerols; PC, phosphatidylcholines; PE, phosphatidylethanolamines; PG, phosphatidylglycerols; PI, phosphatidylinositols; PS, phosphatidylserines; SM, sphingomyelins; ST, sterol lipids; TG, triacylglycerols.

# Figure S7

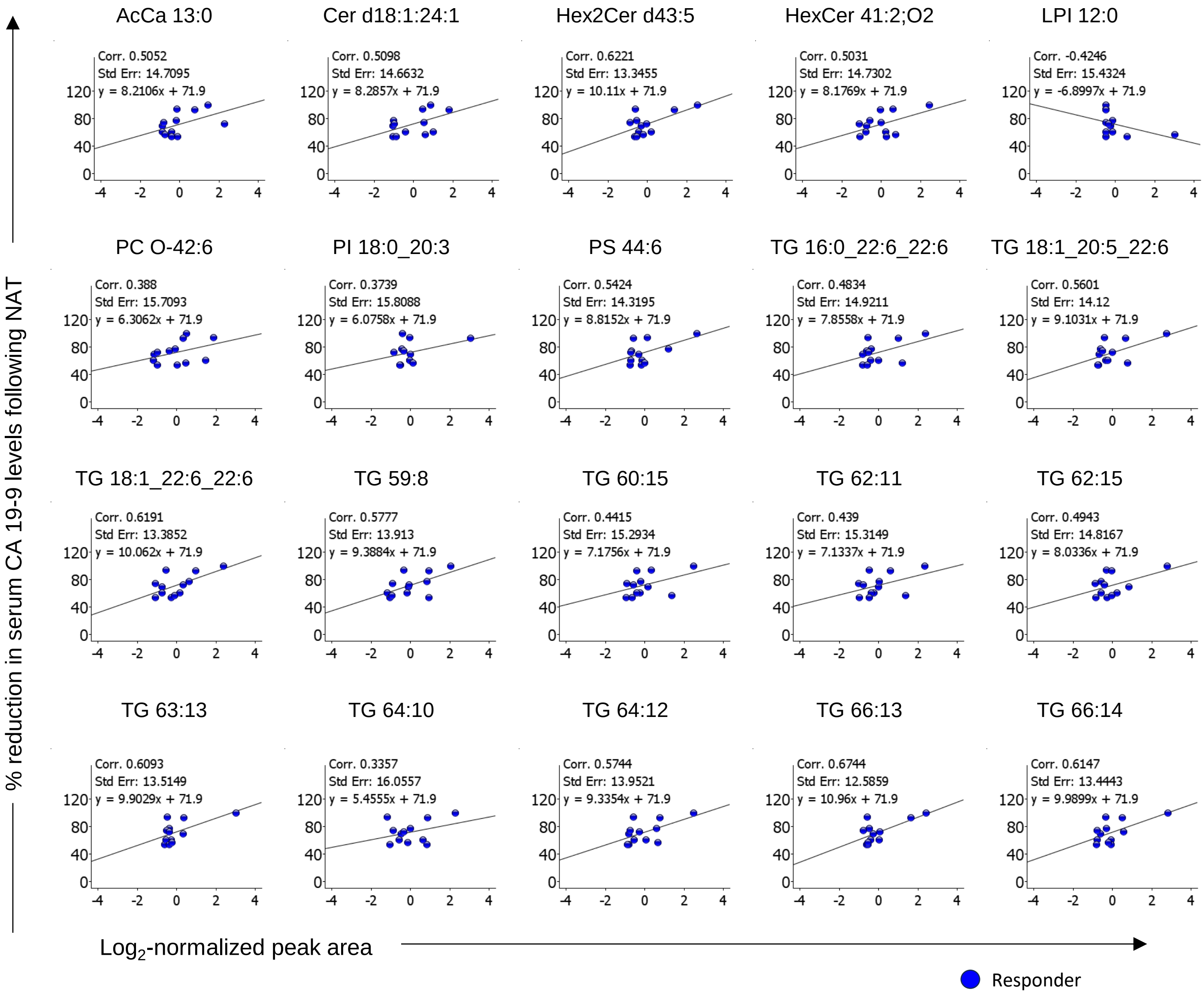


**Fig. S7.** Scatter plots showing correlation between the abundance of serum DALs (X-axis) and percentage reduction in serum CA 19-9 level following neoadjuvant FOLFIRINOX treatment (Y-axis). AcCa, acylcarnitine; CA 19-9, carbohydrate 19-9 antigen; Cer, ceramides; HexCer, hexosylceramides; LPI, lysophosphatidylinositols; PC, phosphatidylcholines; PI, phosphatidylinositols; PS, phosphatidylserines; TG, triacylglycerols.

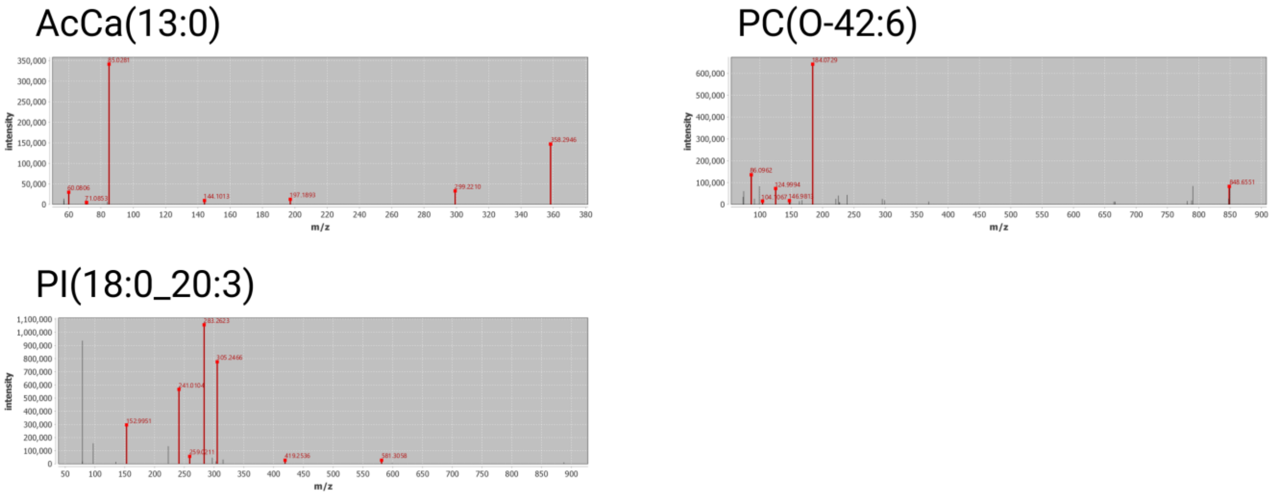


**Fig. S8.** Fragmentation spectra for differentially abundant lipids (DALs) in main Figure 7A. AcCa, acylcarnitine; PC, phosphatidylcholines; PI. phosphatidylinositols.

**Figure S9**

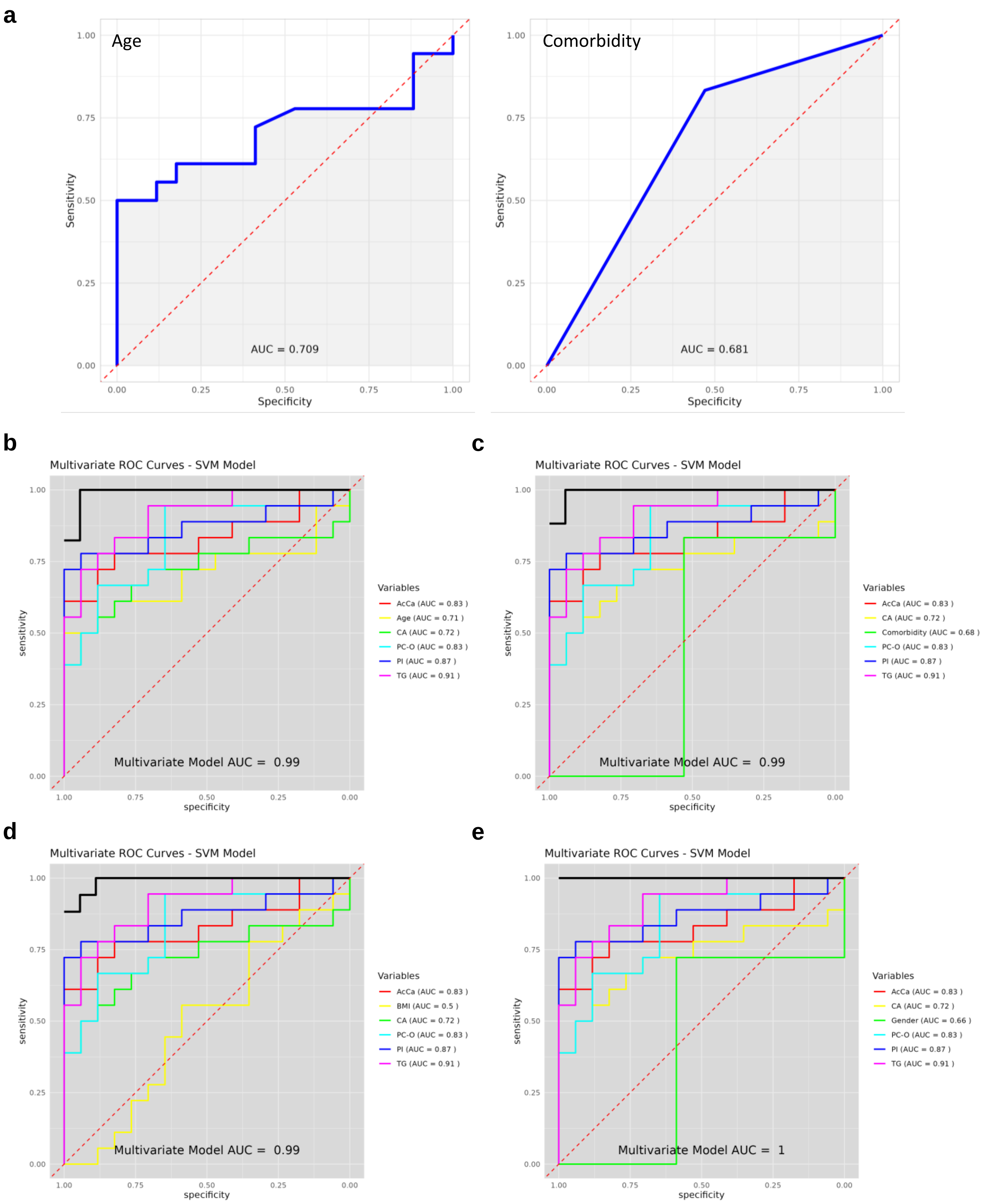


**Fig. S9.** Assessment of diagnostic performance prediction. (**a**) Individual receiver operating characteristic (ROC) curves generated using LipidOne for age and comorbidities. (**b-e**) Multivariate ROC curves computed for five serum DALs plus CA 19-9 combined with (**b**) age, (**c**) comorbidity, (**d**) BMI and (**e**) gender. AUC, area under curve; AcCa, acylcarnitine; BMI, body-mass index; CA, carbohydrate antigen (19-9); PC, phosphatidylcholines; PI, phosphatidylinositols; SVM, support vector machine; TG, triacylglycerols.
