# Supplementary material for: Reduced lipid metabolite abundance in human pancreatic cancer and matched serum samples following neoadjuvant FOLFIRINOX treatment
Source: Metabolomics. 2026 Jan 19;22(1):18. doi: 10.1007/s11306-025-02388-z (PMC12816000; doi:10.1007/s11306-025-02388-z)
Supplement: Supplementary file 3 — Supplementary file3 (PDF 276 KB) [file 11306_2025_2388_MOESM3_ESM.pdf]

Table S1. Clinical characteristics of the study population.

PC ID	Group	Inclusion year	Gender (0=F, 1=M)	Age (Years)	BMI	Type of co-morbidity	Disease stage (resectable)	Specimen type	NAT regimen (FOLFIRINOX, # of cycles)	CA 19-9 (pre-NAT; U/mL)	CA 19-9 (post-op; U/mL)
20	TN	2015	1	50	23,8	-	PRPC	PPPDE	-	-	677
21	TN	2015	1	77	21,7	1+5	PRPC	PDE	-	-	<5
22	TN	2015	0	65	21,5	2+5	PRPC	DPE	-	-	110
31	TN	2017	1	79	22,5	1+2	PRPC	PDE	-	-	1676
34	TN	2017	1	64	26,4	2	PRPC	PPPDE	-	-	299
38	TN	2017	1	70	23,8	2	PRPC	PPPDE	-	-	<5
40	TN	2017	1	76	20,7	-	PRPC	PPPDE	-	-	35
51	TN	2017	1	58	32,0	1+3+5	PRPC	PDE + VMS	-	-	191
53	TN	2017	0	76	39,6	1+3	PRPC	PDE	-	-	282
72	TN	2018	1	55	25,6	1+3	PRPC	PPPDE	-	-	2932
76	TN	2018	1	55	29,3	-	PRPC	PDE	-	-	230
80	TN	2018	0	73	21,8	1+2+3	PRPC	PPPDE	-	-	11371
90	TN	2020	0	78	34,0	3+5	PRPC	DPE	-	-	178
91	TN	2020	1	65	26,6	1	PRPC	PPPDE	-	-	774
101	TN	2021	1	78	23,4	4+5	PRPC	PPPDE	-	-	493
103	TN	2021	0	76	23,0	2+5	PRPC	PDE	-	-	616
110	TN	2021	1	84	23,3	1+2	PRPC	DPE	-	-	11
111	TN	2021	1	81	26,9	2	PRPC	DPE	-	-	70
35	NAT	2016	1	68	17,3	-	BRPC	PDE+VMS	x4	97	30
39	NAT	2016	1	64	25,2	-	BRPC	PDE	x4	-	93
42	NAT	2017	0	60	21,7	-	PRPC	PDE	x4	878	56
43	NAT	2017	1	60	34,8	-	LAPC	TPE + AMS	x7	272	107
52	NAT	2017	1	53	22,4	-	PRPC	PDE	x4	56	13
61	NAT	2017	0	59	22,9	5	PRPC	PDE	x2	8827	19
66	NAT	2018	0	69	19,1	2	BRPC	PDE + VP	x4	15	7
67	NAT	2018	0	68	25,0	3+5	PRPC	PPPDE	x4	31	53
68	NAT	2018	0	66	27,5	3	BRPC	PPPDE	x5	1035	268
70	NAT	2018	1	71	32,1	2+5	BRPC	PDE	x6	365	145
77	NAT	2018	1	62	29,7	1+3	PRPC	PDE	x4	463	477
81	NAT	2018	0	59	23,5	-	PRPC	PPPDE	x4	768	55
100	NAT	2020	0	51	33,3	-	BRPC	PPPDE	x4	73	34
105	NAT	2020	0	75	24,5	1+4+5	BRPC	PPPDE+VMS	x4	-	31
109	NAT	2021	0	59	21,2	-	BRPC	PDE+VMS	x4	85	187
114	NAT	2021	0	64	28,1	5	BRPC	PPPDE	x4	491	213
118	NAT	2022	1	74	24,7	-	BRPC	PDE	x9	289	80

PC ID	Bilirubin (µmol/L)	Albumin (mg/dL)	CRP (mg/L)	Tumor size (mm)	T stage	N stage	TRG	Survival Dec.2023	Survival (months)	Adjuvant regimen
20	22	45	1,2	35	3	2	-	1	7,9	Folfirinox
21	58	36	7,2	55	3	2	-	0	102,9	FLV
22	5	44	1,3	32	3	0	-	1	18,5	-
31	10	44	<0.6	33	3	2	-	1	2,4	-
34	55	39	4,3	29	3	2	-	1	2,4	-
38	34	41	17	35	3	2	-	0	78,9	-
40	8	44	<0,6	28	3	1	-	0	78,9	Gemcitabine
51	16	43	<0,6	38	3	1	-	1	38,8	GemCap
53	29	39	7	42	3	0	-	1	32,3	NA
72	31	43	0,8	36	2	2	-	1	39,0	GemCap
76	107	41	2,2	39	2	1	-	1	5,8	Folfirinox
80	33	40	1,5	60	3	2	-	1	5,7	-
90	9	45	0,9	51	3	1	-	0	40,7	Gemcitabine
91	272	39	24	45	3	2	-	1	22,9	mFolfirinox
101	38	34	19	37	2	1	-	1	9,9	GemCap
103	341	37	14	30	2	0	-	1	7,0	-
110	5	38	30	102	3	0	-	0	29,8	-
111	6	40	0,6	37	2	2	-	1	6,9	-
35	4	29	0,9	36	3	1	CAP3	1	8,1	FLV
39	7	40	3,5	42	3	1	CAP3	1	41,4	GemCap
42	4	44	0,8	28	3	2	CAP2	1	20,1	GemCap
43	12	44	2,3	35	3	2	CAP3	1	9,0	-
52	6	42	17	38	3	1	CAP2	1	16,8	GemCap
61	4	43	4,4	31	2	2	CAP2	1	21,3	GemCap
66	4	42	3,8	30	3	0	CAP3	1	20,0	Gem
67	9	41	2,4	33	3	2	CAP3	1	17,4	GemCap
68	9	40	2,1	36	2	1	CAP2	1	17,8	Folfirinox
70	6	43	3,3	48	3	2	CAP3	1	30,9	GemCap+FLV
77	24	42	2,2	35	2	1	CAP2	1	30,6	Folfirinox
81	4	42	1	22	2	0	CAP2	0	64,2	Folfirinox
100	3	37	2	48	3	1	CAP2	0	37,1	Folfirinox
105	4	41	1	27	2	2	CAP2	1	12,7	Gemcitabine
109	3	39	3	33	2	1	CAP3	1	10,5	GemCap
114	6	44	4,6	23	2	1	CAP3	0	29,9	Folfirinox
118	5	43	11	54	3	2	CAP2	0	12,9	n/a

Comorbidity type: 1= diabetes mellitus, 2=cardiovascular, 3=hypertension, 4= COPD, 5=other. TN, treatment-naïve; NAT, neoadjuvantly treated; BMI, body-mass index; CA 19-9, Carbohydrate 19-9 antigen; CAP, College of American Pathologists; DPE, distal pancreatectomy; FLV, fluorouracil (5-FU) plus leucovorin (LV); PPPDE, pylorus-preserving pancreatoduodenectomy; TPE, total pancreatectomy; TRG, tumor regression grade; PC, pancreatic cancer; PRPC, primary resectable PC; BRPC, borderline resectable PC; LAPC, locally advanced PC.
