# Supplementary material for: Reduced lipid metabolite abundance in human pancreatic cancer and matched serum samples following neoadjuvant FOLFIRINOX treatment
Source: Metabolomics. 2026 Jan 19;22(1):18. doi: 10.1007/s11306-025-02388-z (PMC12816000; doi:10.1007/s11306-025-02388-z)
Supplement: Supplementary file 4 — Supplementary file4 (PDF 144 KB) [file 11306_2025_2388_MOESM4_ESM.pdf]

Table S2. Univariate ROC analysis for individual serum DALs.

Name	Lipid class	AUC	t-test	Log2FC
PI 18:0_20:3	GP	0.8660	0.00146	0.8494
AcCa 13:0	FA	0.8268	0.00012	−1.7721
PC O-42:6	GP	0.8268	0.00051	−1.1326
TG 49:6	GL	0.8072	0.00176	−1.2138
TG 66:14	GL	0.7843	0.00722	−1.9187
TG 18:1_22:6_22:6	GL	0.7811	0.01248	−1.6271
TG 64:10	GL	0.7778	0.03081	−1.0256
CAR 6:1;O2	FA	0.7712	0.00709	−1.0361
HexCer 41:2;O2	SP	0.7712	0.00309	−0.8765
FA 22:1	FA	0.7647	0.00995	−0.9791
TG 18:2_18:4_20:5	GL	0.7614	0.00203	−2.2388
PS 44:6	GP	0.7484	0.00943	−1.6214
Hex2Cer 43:5;O2	SP	0.7386	0.02621	−1.4339
TG 59:8	GL	0.7320	0.03053	−1.0159
TG 66:13	GL	0.7254	0.03336	−1.6228
PEtOH 18:1_26:4	GP	0.7222	0.01932	1.1205
TG 63:13	GL	0.7091	0.03312	−1.7195
FA 12:4;O3	FA	0.6994	0.02704	−1.2315
FA 12:5;O3	FA	0.6961	0.05088	−1.7799
TG 60:15	GL	0.6928	0.03038	−1.4801
TG 62:15	GL	0.6895	0.01297	−1.8221
Cer 18:1;O2_24:1	SP	0.6699	0.05033	1.2119
TG 18:3_20:5_22:6	GL	0.6569	0.01456	−1.7949
CA 19-9	-	0.7222	0.13226	−3.3347
Age	-	0.7091	0.03835	−0.1373
Comrbidity	-	0.6813	0.02356	−0.8244
Gender	-	0.65523	0.06663	−0.8106
BMI	-	0.50163	0.80687	−0.0232

DALs with statistically significant diagnostic performance ($p<0.05$). AUC represents area under the individual ROC curve while Log2FC values represent fold change between NAT versus TN samples. BMI, body-mass index; CA 19-9, carbohydrate 19-9 antigen; Cer, ceramide; DAL, differentially abundant lipid; FA, fatty acids/acyls; GL, glycerolipids; GP, glycerophospholipids; HexCer, hexosylceramide; NAT, neoadjuvant chemotherapy treated; PC, phosphatidylcholine; PI, phosphatidylinositol; PS, phosphatidylserine; ROC, receiver operating characteristic; SP, sphingolipid; TG: triacylglycerol; TN, treatment naïve.
